# Shoot-to-root translocated GmNN1/FT2a triggers nodulation and regulates soybean nitrogen nutrition

**DOI:** 10.1371/journal.pbio.3001739

**Published:** 2022-08-15

**Authors:** Xinxin Li, Huiwen Zhou, Ling Cheng, Niannian Ma, Baofeng Cui, Wenfei Wang, Yongjia Zhong, Hong Liao

**Affiliations:** 1 Root Biology Center, Fujian Agriculture and Forestry University, Fuzhou, China; 2 College of Life Sciences, Fujian Agriculture and Forestry University, Fuzhou, China; UCSD, UNITED STATES

## Abstract

Symbiotic nitrogen fixation (SNF) provides sufficient nitrogen (N) to meet most legume nutrition demands. In return, host plants feed symbionts carbohydrates produced in shoots. However, the molecular dialogue between shoots and symbionts remains largely mysterious. Here, we report the map-based cloning and characterization of a natural variation in *GmNN1*, the ortholog of *Arabidopsis thaliana FLOWERING LOCUS T* (*FT2a*) that simultaneously triggers nodulation in soybean and modulates leaf N nutrition. A 43-bp insertion in the promoter region of *GmNN1/FT2a* significantly decreased its transcription level and yielded N deficiency phenotypes. Manipulating *GmNN1*/*GmFT2a* significantly enhanced soybean nodulation, plant growth, and N nutrition. The near-isogenic lines (NILs) carrying low mRNA abundance alleles of *GmNN1*/*FT2a*, along with stable transgenic soybeans with CRISPR/Cas9 knockouts of *GmNN1*/*FT2a*, had yellower leaves, lower N concentrations, and fewer nodules than wild-type control plants. Grafting together with split-root experiments demonstrated that only shoot GmNN1/FT2a was responsible for regulating nodulation and thereby N nutrition through shoot-to-root translocation, and this process depends on rhizobial infection. After translocating into roots, shoot-derived GmNN1/FT2a was found to interact with GmNFYA-C (nuclear factor-Y subunit A-C) to activate symbiotic signaling through the previously reported GmNFYA-C-ENOD40 module. In short, the description of the critical soybean nodulation regulatory pathway outlined herein sheds novel insights into the shoot-to-root signaling required for communications between host plants and root nodulating symbionts.

## Abstract 2

Symbiotic nitrogen fixation provides a vital nitrogen source in agroecosystems but nodulation is tightly controlled by a long-distance signaling system. This study uses map-based cloning to reveal GmNN1/FT2a as a new shoot-to-root mobile protein that significantly regulates nodule formation and thus nitrogen nutrition in soybean.

## Introduction

With the inclusion of nearly 750 genera and 20,000 species, legumes constitute the third largest family of angiosperms [1]. Most leguminous plants are capable of establishing symbiotic associations with soil-dwelling rhizobia that lead to the formation of nodules where symbiotic nitrogen fixation (SNF) takes place [2]. This association enables leguminous plants to reduce atmospheric N_2_ gas to biologically available ammonia [3]. In this mutual exchange, soybean plants provide rhizobia with carbohydrates derived from photosynthesis in return for nitrogen (N) provided by rhizobia through SNF [4]. It has been reported that the N provided through SNF could cover up to 70% of the total N needed for growth and development of soybean plants, as well as N rich residue for subsequent or intercropped planting [2,5,6].

The mutual symbiosis between legumes and rhizobia involves complicated biochemical dialogue through symbiotic signaling networks [7–11]. To date, several important regulators of symbiotic signaling have been identified, including nodule inception (NIN), nuclear factor-Y subunit A1 (NF-YA1), and nodulation signaling pathway 1 and 2 (NSP1/NSP2) [8,11–13]. Furthermore, SNF is an energy-intensive metabolic process requiring tight control by the host legume to balance the trade-offs between allocation of resources for growth or nodulation [14]. Long distance signaling through autoregulation of nodulation (AON) is one reported pathway employed by legume host shoots to control nodulation in the roots [15–17]. In addition, environmental conditions, such as high N supply, have been reported to significantly inhibit nodulation [10,11,18–20]. Recently, long distance light signaling has also been reported to be involved in the regulation of nodulation through the CCaMK-STF-FT module [17,21]. However, specifics of the molecular dialogue between host shoots and symbionts and the fine tuning of resource division between nodulation and development remain largely mysterious.

In this study, we identified a quantitative trait locus (QTL) and the major gene *GmNN1* in this locus that is responsible for shoot control of root nodulation. We further identified a naturally occurring variant with a 43-bp insertion in the promoter region of *GmNN1* that leads to lower expression of *GmNN1* and inhibits its translocation from shoots to roots. Further observation revealed that the GmNN1/FT2a-GmNFYA-C module regulated nodulation through association with the promoter of *GmENOD40*. Overall, this work elucidates a novel regulatory component in soybean nodulation and provides new insights into communications between host plants and symbionts through shoot-to-root translocated signaling components.

## Results

### Discovery of natural variation in nitrogen nutrition in soybeans

**Fig 1:**
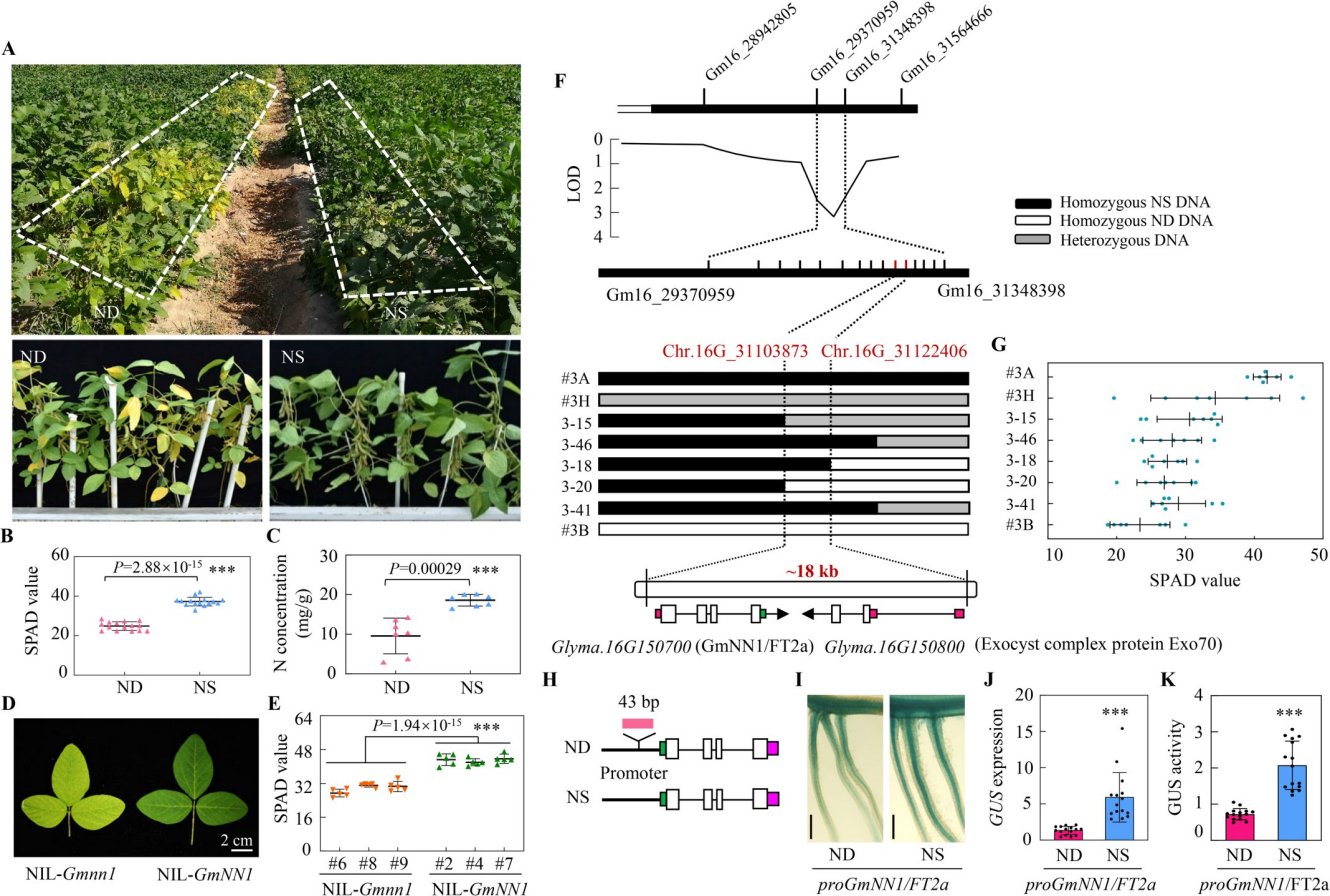
Positional cloning of *qNN1* on chromosome 16. ** (A)** Pictures of ND and NS soybean materials grown in the field**. (B)** SPAD value (*n* = 15). **(C)** Leaf N concentration (*n* = 7). Values are the means from 2 independent experiments. **(D)** Pictures of new leaves from the NILs of *qNN1*. **(E)** Leaf SPAD value of NILs (*n* = 5). Three independent lines of each NIL were generated, and 5 biological replicates for each line were harvested for SPAD value analysis. **(F)** Map-based cloning of *GmNN1*. Fine mapping was performed with sub-F_3:4_ populations. The delimited 18-kb genomic region for *qNN1* contains 2 predicated genes in the reference genome of Ws82. **(G)** Putative phenotypes of the recombinant plants were evaluated based on observations of the progeny (*n* = 7). **(H)** Gene structure and allelic variation of *GmNN1* between ND and NS. Hollow, green, and red boxes represent exons, 3′-UTR and 5′-UTR, respectively. Bold and fine lines represent promoter and intron regions, respectively. **(I)** GUS staining of the soybean transgenic hairy roots harboring the *GmNN1* promoter (*proGmNN1*/*FT2a*) PCR amplified from either ND or NS plant. Scale bars, 500 μM. **(J)** Relative expression of the *GUS* gene (*n* = 15). **(K)** Quantitative GUS activity measured in fluorimetric assays (*n* = 15). All data are given as mean ± SD. Asterisks denote significance of differences (threshold *P* = 0.05) according to Student *t* tests; ***P < 0.001. Data underlying the graphs in the figure can be found in S1 Data. N, nitrogen; ND, N deficient; NIL, near-isogenic line; NS, N sufficient.

In the field, we found 2 genotypes segregating from the same sub-progeny that displayed distinct nitrogen (N) nutrition phenotypes, with the ND (N deficient) line exhibiting yellower leaves than the NS (N sufficient) line (Fig 1A), which was verified by 33.3% and 48.5% lower SPAD values and leaf N concentrations in ND than in NS plants (Fig 1B and 1C). To dissect the genetic basis for this natural variation in leaf N nutrition, we first re-sequenced the ND and NS genotypes. The results showed that only 4 regions on chromosome 16 were highly heterozygous (S1 Fig and S1 Table). To further determine which region is required for controlling N nutrition, we generated a 132 member sub-mapping population that was derived from 5 individual F_5_ residual heterozygous plants. One QTL for N concentration was localized to a region between the Gm16_29370959 and Gm16_31348398 markers located in region 4 and was subsequently named as nitrogen nutrition locus 1 (*qNN1*) (Fig 1F). To validate the role of *qNN1* in controlling N nutrition, we developed the following near isogenic lines (NILs), NIL-*Gmnn1*, which carries the ND allele, and NIL-*GmNN1*, which carries the homologous segment from NS. Phenotyping analysis showed that NIL-*Gmnn1* plants have yellower leaves and lower SPAD values than NIL-*GmNN1* lines (Fig 1D and 1E).

### Map-based cloning of *GmNN1*

Fine mapping narrowed down the location of *qNN1* to an interval between the 2 markers Chr.16G_31103873 and Chr.16G_31122406 containing 2 putative genes, *Glyma*.*16G150700* and *Glyma*.*16G150800* (Fig 1F). Phenotype of the recombinant plants were also evaluated in progeny lines. Consistently, #3B lines harboring the ND allele had lower SPAD values than #3A lines harboring the NS allele and progeny lines exhibited large variations in SPAD values (Fig 1G). We then sequenced these 2 genes in both ND and NS plants. In comparison with NS plants, the *Glyma*.*16G150700* (also known as Flowering Locus T, *FT2a*) gene in ND contains one 43-bp deletion in the promoter region (Fig 1H), while *Glyma*.*16G150800* encodes an exocyst complex protein not displaying any variation between NS and ND plants. Therefore, we considered *Glyma*.*16G150700* as the causal gene underlying phenotypic variation associated with *qNN1*, and hereafter the gene was renamed *GmNN1/FT2a*.

To investigate whether the 43-bp variation in the promoter of *GmNN1/FT2a* is critical for gene expression, the isolated promoters of ND and NS were fused to separate *GUS* reporter genes and then transferred to both hairy roots of transgenic composite soybean plants and tobacco leaves (Figs 1I and S2A). Although *GmNN1/FT2a* promoters were able to drive *GUS* gene expression, transgenic hairy roots and tobacco leaves harboring *proGmNN1/FT2a*::*GUS* amplified from ND plants exhibited lower GUS activity than those harboring constructs from the NS genotype. The expression level of *GUS* and its protein activity mediated by *GmNN1/FT2a* from NS were 4.39-fold and 2.86-fold in hairy roots and 2.84-fold and 4.31-fold higher in transient transgenic tobacco compared with the impacts of *GmNN1/FT2a* from ND (Figs 1J, 1K, S2B, and S2C). Moreover, *GmNN1/FT2a* transcription was also evaluated in NILs, with *GmNN1/FT2a* transcript levels significantly higher in NIL-*GmNN1* lines than in NIL-*Gmnn1* plants (S2D Fig). These results suggest that variation in the promoter region of *GmNN1/FT2a* leads to differences in mRNA accumulation that might be responsible for the improved N nutrition in NS relative to ND soybean plants.

### Manipulating GmNN1/GmFT2a significantly impacts nodulation and N nutrition in soybean

**Fig 2:**
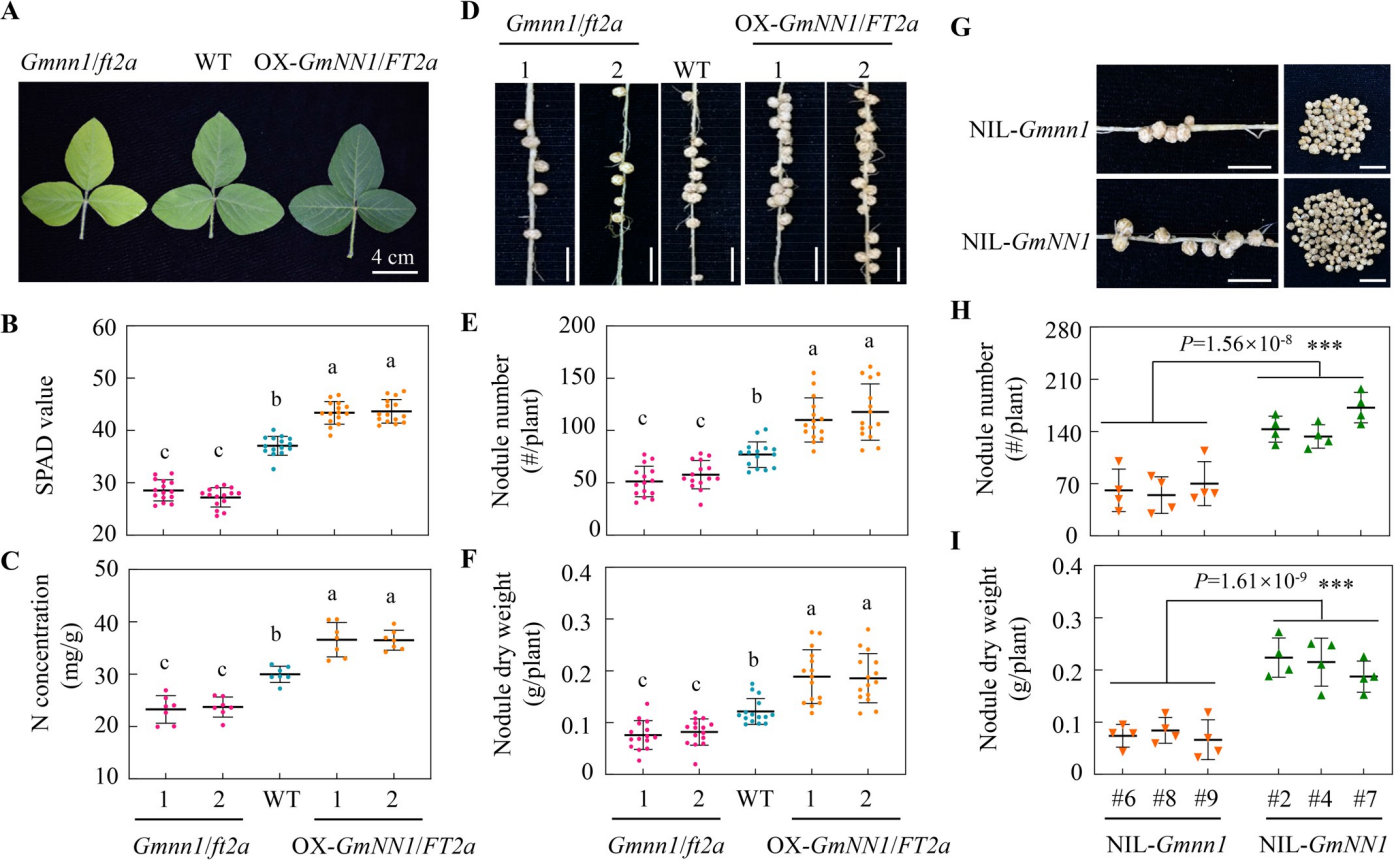
Functions of *GmNN1/FT2a* in nitrogen nutrition and nodulation. **(A**) Pictures of new leaves at 21 dai in loss-of-function mutants of *GmNN1/FT2a* (*Gmnn1*/*ft2a*), WT, and *GmNN1/FT2a* overexpressing (OX-*GmNN1/FT2a*) lines. **(B)** Leaf SPAD values (*n* = 15). **(C**) Leaf N concentration (*n* = 7). Values are the means from 2 independent experiments. **(D**) Phenotype of nodulation. Scale bars, 1 cm. **(E**) Nodule number (*n* = 15). (**F**) Nodule dry weight per plant (*n* = 15). **(G)** Performance of nodulation at 20 dai between NIL-*Gmnn1* and NIL-*GmNN1*. Scale bars, 1cm. **(H)** Nodule number (*n* = 4). **(I)** Nodule dry weight per plant (*n* = 4). Three independent lines of each NIL were generated and 4 biological replicates for each line were harvested for nodulation analysis. All data are given as mean ± SD. Different letters denote significant differences (*P* < 0.05) according to Duncan’s multiple range comparison tests, and asterisks denote significance of differences (threshold *P* = 0.05) according to Student *t* tests; ***P < 0.001. Data underlying the graphs in the figure can be found in S1 Data. dai, day after inoculation; NIL, near-isogenic line; WT, wild type.

To further outline potential roles filled by GmNN1/FT2a in soybean N nutrition, we also generated stable transgenic soybeans in the Williams 82 (Ws82, wild type, WT) background carrying the NS allele, including 2 independent CRISPR/Cas9 *GmNN1/FT2a* knockout lines (*Gmnn1/ft2a*, S3 Fig), as well as 2 independent lines overexpressing *GmNN1/FT2a* (OX-*GmNN1/FT2a*, S4 Fig). The 2 knockout lines exhibited obviously delayed flowering (S4A and S4C Fig) and yellower leaves than WT plants, while OX-*GmNN1/FT2a* lines transcribing *GmNN1/FT2a* 12.1- to 16.6-fold higher than WT displayed opposite phenotypes (Figs 2A and S4B). The SPAD value decreased by 23.0% and 26.6% in the 2 *Gmnn1/ft2a* mutants and increased by 16.9% and 17.7% in the OX-*GmNN1/FT2a* lines (Fig 2B). Meanwhile, leaf N concentrations decreased by averages of 22.4% and 20.8% in knockout mutants and increased by 22.1% and 21.7% in OX-*GmNN1/FT2a* lines (Fig 2C).

Since SNF is a vitally important and irreplaceable N source for legumes, nodulation traits for WT and mutant lines were therefore investigated in hydroponics at 21 d after rhizobium inoculation (dai). Intriguingly, we found that manipulating *GmNN1/GmFT2a* significantly affected soybean nodulation, and as shown in Fig 2D, nodules were less numerous in mutants and more numerous in OX-*GmNN1/FT2a* lines in comparisons with each other and with WT plants. Nodule counts declined by 42.9% and 32.9%, and nodule weights decreased by 37.6% and 32.7% in knockout lines compared with WT plants. Conversely, overexpression of *GmNN1/FT2a* led to 43.0% and 52.9% increases in nodule numbers, accompanied by 54.9% and 52.6% increases in nodule biomass relative to nodulation on WT plants (Fig 2E and 2F). This strong genetic component was also confirmed in NILs, as NIL-*Gmnn1* lines displayed fewer nodules and lower nodule dry weights than NIL-GmNN1 lines (Fig 2G–2I). In addition, the nodulation characteristics of ND and NS genotypes were further evaluated in hydroponics after rhizobium inoculation. Consistent with the observations in mutant lines and naturally occurring variants outlined above, ND plants with yellow leaf phenotypes harbored fewer nodules (S5A Fig). The total number of nodules was 36.4% lower in ND lines than in NS lines (S5B Fig), with concomitant nodule dry weight losses totaling 41.0% (S5C Fig). Taken together, these results suggest that GmNN1/FT2a simultaneously triggers soybean leaf N limitation responses and nodulation on roots.

### Manipulating GmNN1/GmFT2a regulates plant growth, nodulation, and N content

**Fig 3:**
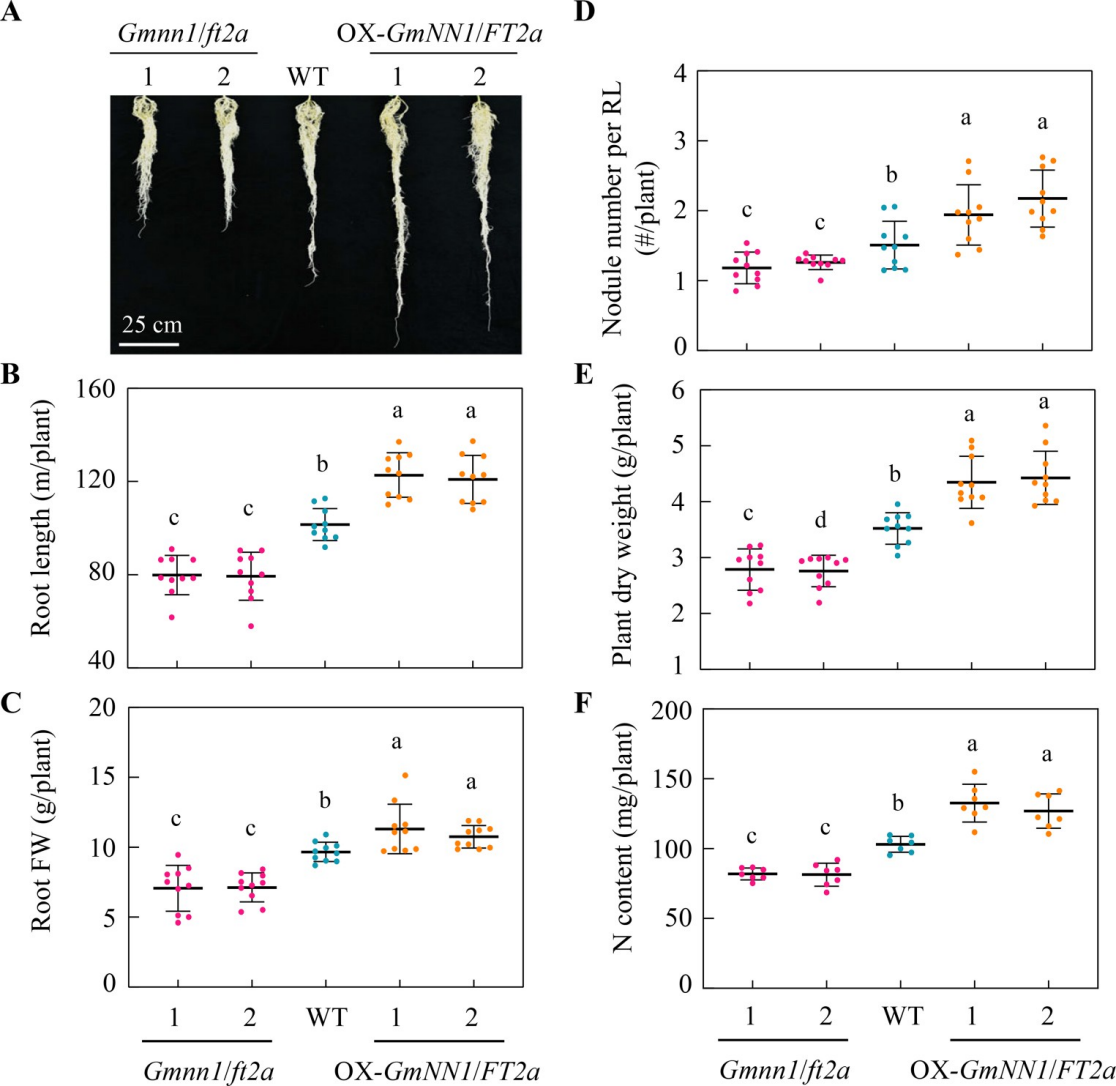
Plant growth, nodulation, and N content as affected by manipulating of GmNN1/FT2a. **(A)** Photographs showing soybean root growth performance. **(B)** Total root length (*n* = 10). **(C)** Root FW (*n* = 10). **(D)** Nodule number per RL (*n* = 10). **(E)** Plant dry weight (*n* = 10). **(F)** N content (*n* = 7). WT and transgenic soybean lines with knockout or overexpression of *GmNN1/FT2a* inoculated with rhizobia were grown in hydroponic for 21dai. All data are given as mean ± SD. Different letters denote significant differences (*P* < 0.05) according to Duncan’s multiple range comparison tests. Data underlying the graphs in the figure can be found in S1 Data. dai, day after inoculation; FW, fresh weight; N, nitrogen; RL, root length; WT, wild type.

To evaluate the effects of *GmNN1/FT2a* on plant growth and N nutrition at the whole plant level, 21 dai roots and shoots were separately harvested for further measurement. As shown in Fig 3A, knockout of *GmNN1/FT2a* significantly inhibited root growth, while OX of *GmNN1/FT2a* obviously promoted root elongation when compared with WT plants. The total root length and root fresh weight decreased by 21.3% to 21.9% and 26.2% to 26.9% in *GmNN1/FT2a* knockout lines and increased by 19.1% to 20.9% and 11.3% to 17.1% in OX lines compared with WT, respectively (Fig 3B and 3C). Since root growth was modified by *GmNN1/FT2a*, nodule number per root length was further analyzed and found that manipulating GmNN1/GmFT2a increased or decreased nodule number per root length 23.1% to 44.1% or 16.4% to 21.6% in overexpressions or knockout mutants, respectively (Fig 3D), suggesting that GmNN1/FT2a is directly involved in nodulation beyond regulating root growth. Moreover, compared with WT lines, overexpression or knockout of *GmNN1/FT2a* led to 20.8% to 21.5% and 20.6% to 21.1% decrease or 23.4% to 38.2% and 23.1% to 28.6% increase in plant dry weight and N content, respectively (Fig 3E and 3F). Taken together, these results suggest that GmNN1/FT2a is indeed an important contributor to plant growth, nodulation, and N nutrition in soybean.

### GmNN1/FT2a impacts nodulation and N nutrition through shoot-to-root translocation

**Fig 4:**
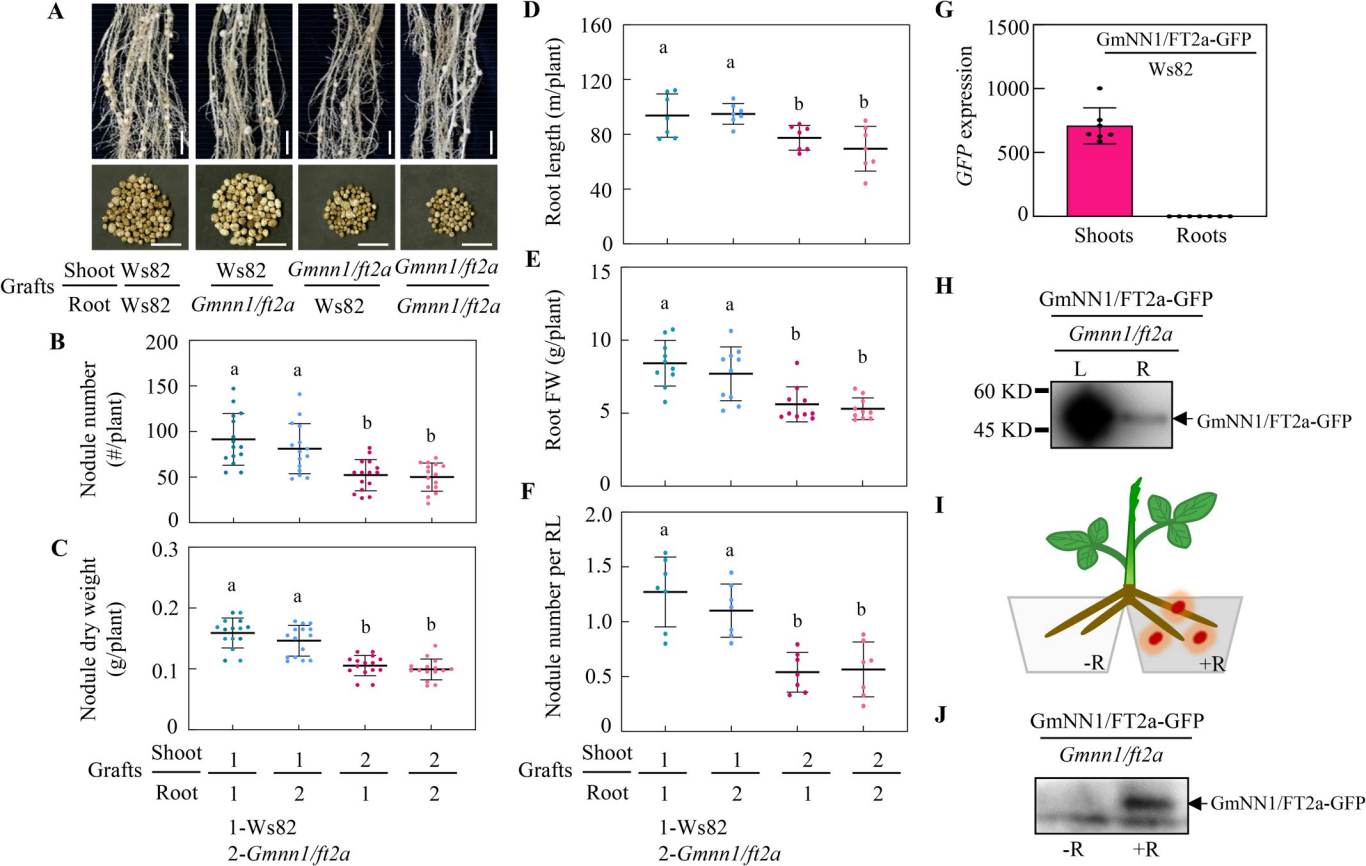
Shoot-to-root translocation of GmNN1/FT2a regulates soybean nodulation. **(A)** Phenotype of nodulation from reciprocal grafting between Ws82 and *Gmnn1*/*ft2a* mutant plants. Scale bars, 2 cm. **(B)** Nodule number from grafts (*n* = 15). **(C)** Nodule dry weight (*n* = 15). Values are the means from 2 independent experiments. **(D)** Root length (*n* = 7). **(E)** Root FW (*n* = 10). **(F)** Nodule number per RL (*n* = 7). The reciprocal grafting plants were harvested at 21 dai. **(G)** Transcript abundance of *GmNN1/FT2a-GFP* in shoot-root grafts at 5 dai (*n* = 7). GmNN1/FT2a-GFP: stable transgenic soybean; Ws82, WT control. **(H)** Immunological detection of GmNN1/FT2a-GFP in leaves (L) and roots (R) of grafts at 5 dai. Scions and rootstocks were stable transgenic soybean plants harboring GmNN1/FT2a-GFP and a knockout of *GmNN1/FT2a* (*Gmnn1*/*ft2a*), respectively. **(I**) Diagram of split-root experiment. **(J**) Immunological detection of GmNN1/FT2a-GFP from split roots 5 dai with (+R) or without (−R) rhizobia. All data are given as mean ± SD. Different letters denote significant differences (*P* < 0.05) according to Duncan’s multiple range comparison tests. Data underlying the graphs in the figure can be found in S1 Data. dai, day after inoculation; FW, fresh weight; RL, root length.

Previous studies revealed that FT products are synthesized in leaves and then travel through the vasculature to the shoot apical meristem to trigger flowering [22]. In this study, we also investigated the expression pattern of *GmNN1/FT2a* and found that transcripts of *GmNN1/FT2a* localized primarily in leaves rather than stems, roots, and nodules (S6 Fig). To further investigate whether shoot-derived GmNN1/FT2a is truly able to systemically enhance nodulation in roots, we also conducted an experiment of reciprocal grafting between WT (Ws82) and *Gmnn1/ft2a* mutant soybeans. Knockout of *GmNN1/FT2a* in scions led to significant decreases in nodule number and nodule dry weight on either WT or *Gmnn1/ft2a* rootstocks (Fig 4A–4C). Moreover, scions knockout of *GmNN1/FT2a* significantly reduced root length, root fresh weight, nodule number per root length, plant dry weight as well as N content in the WT rootstocks to those equivalent to *Gmnn1/ft2a* rootstocks (Figs 4D–4F and S7). In contrast, grafting between WT scions and *Gmnn1/ft2a* rootstocks did not produce significant effects on nodulation traits, plant growth, and N content compared with self-grafted WT plants. Taken together, grafting results suggest that shoot GmNN1/FT2a systemically restores nodule formation and root growth capabilities to *Gmnn1/ft2a* roots.

Another grafting experiment was further carried out to determine whether GmNN1/FT2a itself functions as the long distance signal moving from shoot to root to mediate soybean nodulation. Transcripts of *GmNN1/FT2a-GFP* were detected only in shoots, but not in roots through quantitative reverse transcription PCR (qRT-PCR) analysis of GmNN1/FT2a-GFP/Ws82 grafts (Fig 4G). Further immunoprecipitation and immunoblot testing revealed that GmNN1/FT2a-GFP products in both shoots and roots of GmNN1/FT2a-GFP/*Gmnn1/ft2a* grafts (Fig 4H). These results suggest that the GmNN1/FT2a protein might be moved from shoots to roots. Additionally, to dissect the relationship between GmNN1/FT2a mobility and rhizobial infection, we established a split-root hydroponic setup, and 1 side of the split roots was inoculated with rhizobia (+R) while the other side without inoculation (−R) (Fig 4I). Then, 5 dai roots were separately harvested for immunoprecipitation and immunoblotting analysis. Surprisingly, the GmNN1/FT2a-GFP signal was only detected in roots with inoculated with rhizobium (Fig 4J), suggesting that shoot-to-root translocation of GmNN1/FT2a depends on rhizobial infection. Collectively, these results paint GmNN1/FT2a as a mobile shoot-derived signal directed to nodulating roots where it plays critical roles in nodule development.

### Shoot-derived GmNN1/FT2a modulates nodulation through symbiotic signaling pathway genes

**Fig 5:**
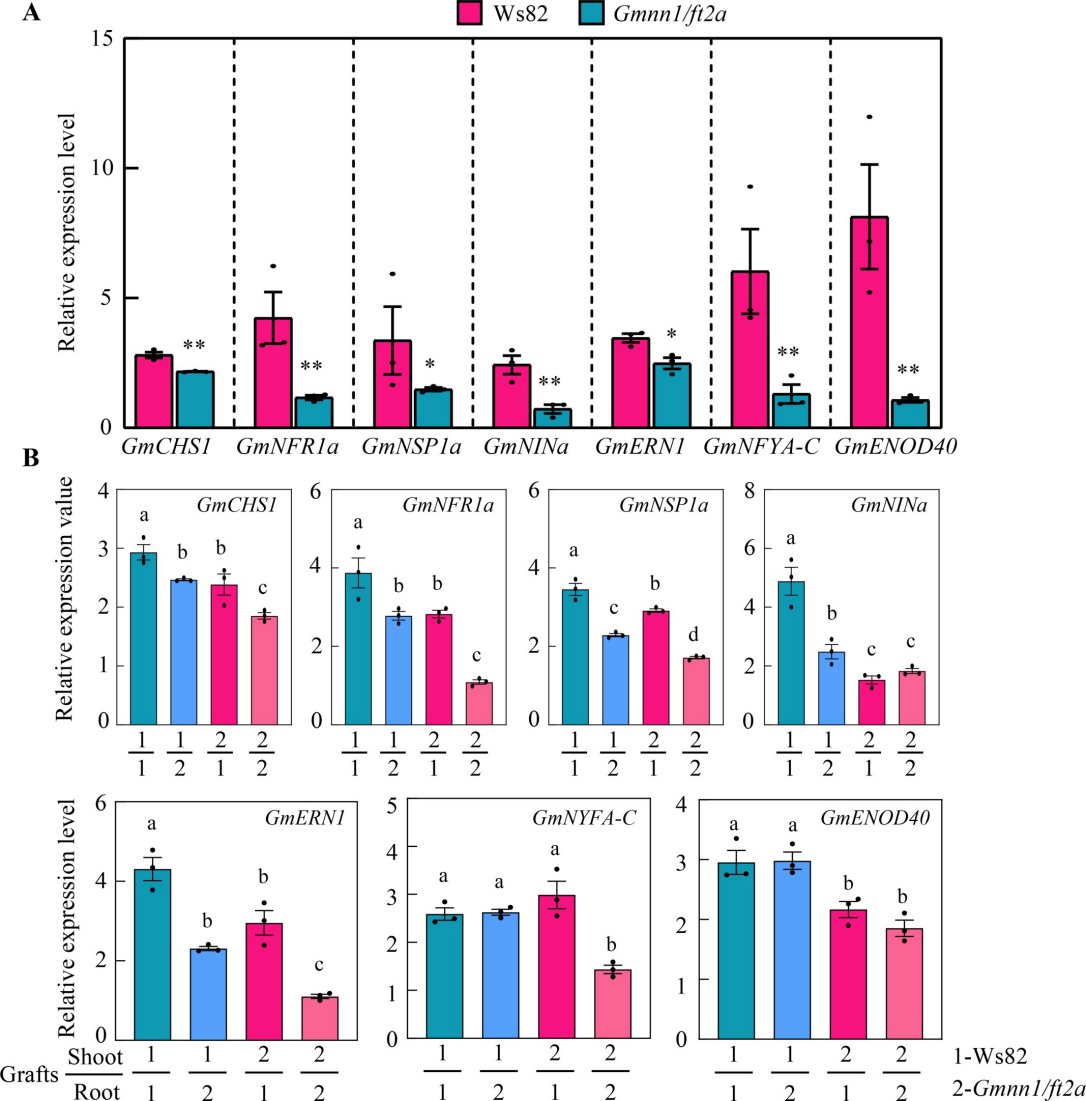
Relative expression levels of symbiotic marker genes in the roots of different plant materials. **(A)** The mRNA abundance of marker genes in WT (Ws82) and *GmNN1/FT2a* knockout mutant (*Gmnn1*/*ft2a*) plants. **(B)** Relative expression of marker genes in the roots of reciprocal grafting treatments conducted between Ws82 and *Gmnn1*/*ft2a*. All data were acquired by qRT-PCR of roots at 3 dai. Date are given as means ± SEM (*n* = 3 technical repeats). Three biological replications were performed with similar results. Data underlying the graphs in the figure can be found in S1 Data. dai, day after inoculation; qRT-PCR, quantitative reverse transcription PCR; WT, wild type.

To investigate molecular mechanisms underlying the GmNN1/FT2a-mediated regulation of nodulation, we first checked for differential expression of symbiotic signal pathway genes between ungrafted Ws82 (WT) and *Gmnn1/ft2a* mutant plants at 3 dai. In these observations, knockout of *GmNN1/FT2a* inhibited most examined marker genes in symbiotic signaling pathway, including *GmCHS1* (Chalcone synthase 1), *GmNFR1*, *GmNSP1a*, *GmNINa*, *GmERN1* (require for nodulation), *GmNYFA-C*, and *GmENOD40* (Fig 5A). Among them, transcripts of *GmNYFA-C* and *GmENOD40* were 4.61 and 7.57 times lower in *Gmnn1/ft2a* mutants than in Ws82 plants. Then, GmNN1/FT2a impacts on the root expression of symbiotic marker genes were verified in reciprocally grafting treatments using Ws82 (WT) and *Gmnn1/ft2a* mutant roots and scions (Fig 5B). In general, the expression level of symbiotic genes was severely inhibited in root when grafting included *Gmnn1/ft2a* in either the scion or rootstock, though impacts tended to be even declined with rootstock expression. The most notable exception was that *GmENOD40* expression was only inhibited with shoot expression of *Gmnn1/ft2a* (Fig 5B), suggesting that shoot-derived *GmNN1/FT2a* stimulates the expression of *GmENOD40*.

**Fig 6:**
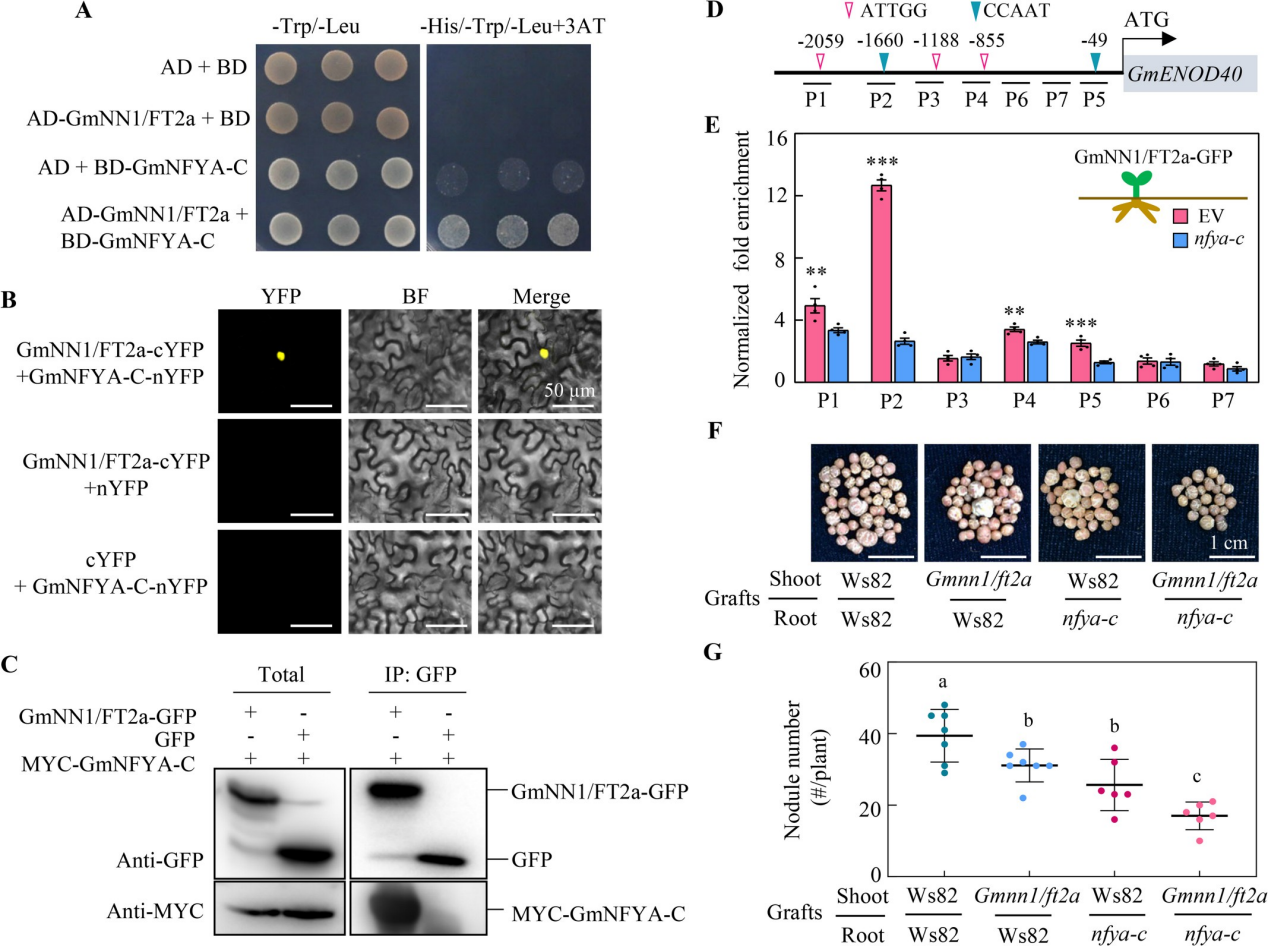
The GmNN1/FT2a-GmNFYA-C-GmENOD40 module regulates soybean nodulation. **(A)** Interaction between GmNN1/FT2a and NYFA-C in the yeast 2-hybrid assay. **(B)** GmNN1/FT2a and GmNFYA-C interaction in the nucleus of tobacco epidermal cells subjected to BiFC analysis. **(C)** GmNN1/FT2a interacting with GmNFYA-C in vitro pull-down assays. **(D)** Diagram of *GmENOD40* promoter. The inverted triangles indicate binding sites of GmNFYA-C. P1 to P7 are the fragments selected for ChIP-qPCR. **(E)** ChIP assay of fragments in the promoter region of *GmENOD40* (*n* = 4). Soybean transgenic composite plants inoculated with rhizobia were grown in sand culture conditions for 5 dai. Hairy roots harboring EV or *nfya-c* mutant produced by CRISPR/Cas9 were separately collected for fixation. **(F and G)** Nodule performance and nodule number of grafts. Nodules were harvested at 21 dai. *Gmnn1*/*ft2a* and *nfya-c*: stable transgenic soybean with knockout of *GmNN1/FT2a* and *GmNFYA-C*, respectively; Ws82: WT control. Data are given as mean ± SD (*n* = 6–7). Different letters denote significant differences (*P* < 0.05) according to Duncan’s multiple range comparison tests. Data underlying the graphs in the figure can be found in S1 Data. BF, bright field; ChIP, chromatin immunoprecipitation; dai, day after inoculation; EV, empty vector; Merge, overlay of the YFP and bright field images; WT, wild type.

The results reported here verify previous work in which GmNFYA-C-GmENOD40 was reported as an important symbiotic signaling molecule [11]. Taken together with the present results showing that shoot-derived *GmNN1/FT2a* stimulates *GmENOD40* expression (Fig 5), we then investigated the possibility of whether GmNN1/FT2a could interact with GmNFYA-C in triggering soybean nodulation. Interestingly, the results revealed a direct interaction between GmNN1/FT2a and GmNFYA-C via a yeast 2-hybrid assay (Fig 6A), which was further confirmed in a bimolecular fluorescence complementation (BiFC) assay (Figs 6B and S8) and co-immunoprecipitation (Co-IP) assays using tobacco leaves co-transformed with GmNN1/FT2a-GFP and MYC-NFYA-C (Fig 6C). To test whether the GmNN1/FT2a-GmNFYA-C module directs the expression of *GmENOD40*, we performed chromatin immunoprecipitation (Ch-IP)-qPCR assays using the transgenic soybean composite plants, consisting of a GmNN1/FT2a-GFP shoot with or without *GmNFYA-C* knockout transgenic hairy roots (Figs 6D, 6E and S9) After 5 dai, the positive hairy roots were separately harvested for ChIP-qPCR analysis, and the results revealed that GmNN1/FT2a-GmNFYA-C regulated the expression of *GmENOD40* through associating with P1, P2, P4, and P5 regions in the promoter, which harbored the canonical *CCAAT* binding site for GmNFYA protein [23]. All of these results are consistent with the conclusion that GmNN1/FT2a directly interact with GmNFYA-C.

**Fig 7:**
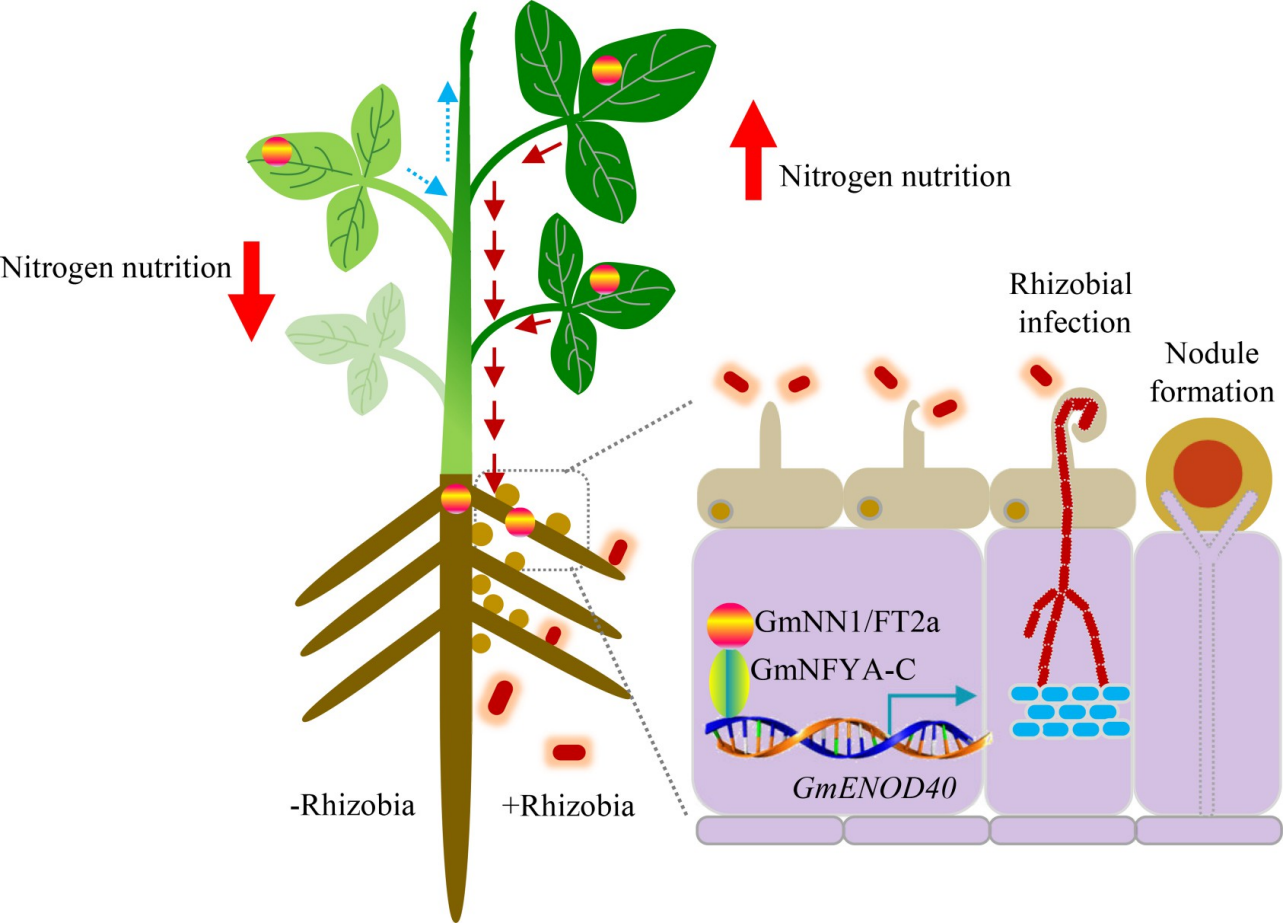
A proposed working model of how the GmNN1/FT2a-GmNYFA-C-GmENOD40 module regulates nodulation in soybean. Shoot-derived GmNN1/FT2a translocates to roots, with its mobility being determined by rhizobial inoculation. In soybean roots, GmNN1/FT2a directly interacts with GmNFYA-C to promote nodulation, and thereby facilitate N nutrition, via activation of *GmENOD40* expression.

More conclusively, using hypocotyl graft chimeras, we observed that in comparisons with Ws82 self-grafted controls, nodule numbers and dry weights declined significantly with grafting between *Gmnn1/ft2a* and Ws82, or between Ws82 and *nfya-c*, and dropped even further with grafting between *Gmnn1/ft2a* scions and *nfya-c* rootsocks (Figs 6F, 6G and S10). These biochemical and genetic results conclusively revealed that GmNN1/FT2a interacts with GmNFYA-C to regulate nodulation through activating the GmENOD40 symbiotic signaling pathway (Fig 7).

## Discussion

This work uncovered natural variation in the promoter region of *GmNN1/FT2a* and further identified the novel regulatory module *GmNN1/FT2a-GmNYFA-C-GmENOD40* as a key element in shoot regulation of root nodulation in soybean plants (Fig 7). To accomplish this, we first bred progeny populations from residual heterozygous lines exhibiting contrasting nitrogen (N) nutrition phenotypes under field conditions (Fig 1A and 1F). Then, we identified a segregating locus and its candidate gene *Glyma*.*16G150700*, which is homologous to *FT* genes (Figs 1F, 1G and S1 and S1 Table) [24]. Further experimentation demonstrated that the observed N deficiency phenotype resulted from a decline in SNF due to a significant decrease in nodulation (Fig 2). This is consistent with previous reports in which nodulation has been tightly associated with N status in soybeans [2,18,25]. Further detailed analysis herein delimited differential expression effects of *Gmnn1/ft2a* versus *GmNN1/FT2a* to a 43-bp insertion in the promoter region of *Gmnn1/ft2a* that significantly inhibited expression (Figs 1H–1K and S2). In addition, overexpression of *GmNN1/FT2a* increased nodulation and simultaneously increased plant growth as well as N nutrition, while knockout of *GmNN1/FT2a* by CRISPR/Cas9 decreased nodulation, plant growth, and N nutrition (Figs 2 and 3). This suggests that N supplied through SNF might be manipulated through altering the expression of *GmNN1/FT2a*. Therefore, the natural variation in *GmNN1/FT2a* discovered presently could be useful for breeding elite cultivars with high SNF capacities.

It has long been known that *GmNN1/FT2a* is mainly expressed in leaves and that it encodes a mobile polypeptide involved in the regulation of the transition to flowering [22,26]. In the present study, we demonstrate alternative functionality in which nodulation on roots appears to be primarily controlled by shoot-to-root translocated GmNN1/FT2a, but not derived from root (Fig 4G and 4H). This is consistent with a previous report that FT regulates the formation of potato tubers [27], as well as a recent publication in which GmNN1/FT2a was found to be translocated from leaves to regulate nodulation in soybean roots [17]. Here, we also demonstrated that the translocation of GmNN1/FT2a depends on rhizobium infection, with GmNN1/FT2a specifically accumulating in the rhizobium infected half of split-root assays (Fig 4I and 4J). We presume that the translocation of GmNN1/FT2a is regulated by an unidentified signal originating from rhizobium infection sites. Other researchers have reported that aboveground systematic signals could be equally distributed among roots [28], so it remains possible that the GmNN1/FT2a translocated to uninfected roots might be degraded by unknown factors. However, the lack of information currently available on the stability of FT protein in planta indicates that these hypotheses require further investigation to sort out [29].

Previous reports have proposed working models with GmNN1/FT2a acting mainly through associations with transcription factors [24]. Here, we demonstrated that GmNN1/FT2a associates with GmNFYA-C (Fig 6A–6E), which has been identified along with homologous genes in other species as essential for nodule initiation and development in *Medicago truncatula*, *Lotus japonicus*, and *Glycine max* [11,30,31]. Moreover, *GmNFYA-C* and *GmNN1/FT2a* exhibited additive effects in the regulation of nodulation, as the number of nodules declined in grafting assays using *Gmnn1/ft2a* and *nfya-c* mutants (Figs 6F, 6G and S10), though redundancy effects of other NYFAs or FTs might obscure some impacts [31,32].

Symbiotic signaling was significantly repressed in the *Gmnn1/fa2a* mutant, which was reversed for specific symbiotic signaling markers by shoot-derived GmNN1/FT2a (Fig 5). This is consistent with previous reports of grafting effects on nodulation (Fig 4A–4C) and further validates the hypothesis that nodulation is controlled largely by shoot-derived GmNN1/FT2a. In this study, upon translocation from shoots to roots, GmNN1/FT2a was found to directly interact with GmNFYA-C (Fig 6A–6E) and then to promote *GmENOD40* (Fig 5), which is a direct target of GmNFYA-C [11]. The expression of *GmNFYA-C* was demonstrated previously to be specifically induced by rhizobial inoculation under low N conditions [11], the interaction between GmNN1/FT2a and GmNFYA-C proved that the regulation of GmNN1/FT2a in nodulation and N nutrition through shoot-to-root translocation is dependent on rhizobial infection and also affected by N conditions. Finally, the expression levels of other symbiotic signaling genes were also influenced in the *Gmnn1/ft2a* mutant, but not through shoot dependent translocation, indicating that *GmNN1/FT2a* could also regulate nodulation through other pathways [17].

Taken together, our findings identify a novel regulatory component in soybean nodulation and provide new insights into communications between host plants and symbionts through shoot-to-root translocated signaling elements.

## Materials and methods

### Plant materials and growth conditions

Experiments in this study included plants grown in the field, pot cultures, and hydroponics. For the field experiments, ND and NS plants were identified at the Dishang experimental farm (E 114.48°, N 38.03°) of the Institute of Cereal and Oil Crops, Hebei Academy of Agricultural and Forestry Sciences, Shijiazhuang City, Hebei Province.

For the pot experiments, seeds of ND and NS lines, as well as seeds from a 132 member sub-mapping population were germinated in pots filled with soil inoculated with the rhizobium strain *Bradyrhizobium* sp. BXYD3 (OD_600_ = 0.8). Seedlings were fertilized once per month with 530 μM low-N nutrient solution as described previously [33]. At the seed-filling stage, plants were harvested to analyze SPAD values, measure N concentrations, and conduct resequencing. Two independent experiments were performed and 15 replicates were investigated for SPAD value, and 7 plants were harvested for N concentration measurement.

For the hydroponic experiment, seeds of soybean used for phenotypic analysis were surface sterilized in 3% H_2_O_2_ for 1 min, rinsed with distilled water, and germinated in sand for 7 d. Uniform seedlings were inoculated with BXYD3 by immersing roots in a rhizobial suspension (OD_600_ = 0.8) for 1.5 h, prior to transplanting into a low-N nutrient solution containing 530 μM N as described above. Plants were grown in growth chambers (day/night: 12 h/12 h, 26°C/24°C). Nutrient solution was refreshed weekly, and the pH was maintained at 5.8 to 6 with diluted H_2_SO_4_ or KOH.

For the grafting assay, plants were sown on sand culture for 4 d. Uniform seedlings were cut at the basal hypocotyls, and scions were grafted on rootstocks. Grafted plants were grown in hydroponics cultures containing 530 μM low-N nutrient solution for 3 d in darkness and then 5 d under light/dark conditions. Subsequently, grafted plants were inoculated with BXYD3 (OD_600_ = 0.8) for 1.5 h as described above. For split-root grafts, part of the main root system was separated and after another 2 d of recovery, 1 side was inoculated with BXYD3 and the other side without inoculation was used as the control. At 5 dai, roots and shoots of grafts were harvested for qRT-PCR, immunoprecipitation (IP), and immunoblotting analysis. At 21 dai, nodules were harvested for phenotyping analysis. Sampled roots were scanned as digital images using a specialized color scanner. Total root length was quantified using a computer image program.

### Resequencing of ND and NS plants

ND and NS were grown in pots and on day 15 of growth, entirely expanded young leaves were harvested. DNA was isolated using a DNeasy Plant Mini Kit. Resequencing libraries were quantified using Bioanalyzer and then sequenced (paired-end, 100-mer each) in the Illumina genome analyzer.

### QTL analysis

Since 4 highly heterozygous sites were found in ND and NS, and to isolate the gene responsible for N nutrition, we separately searched F_5_ residual heterozygous plants derived from the parents of ND and NS. Then, QTL analysis was carried out in MapQTL 6.0 (https://mybiosoftware.com/) to further narrow down the position. An LOD threshold score of 2.5 was considered as the minimum necessary to declare that given QTLs were significant. Among them, we identified a QTL for N concentration in leaves named as *qNN1* (N nutrition locus1) on chromosome 16, which was close to site 4 (S1 Fig) identified in the 132 member sub-mapping population.

### Positional cloning of *qNN1*

A line derived from the mapping population displaying heterozygosity at the *qNN1* locus was selected for fine mapping of *qNN1*. The genes of interest were narrowed down to Gm16_29370959 and Gm16_31348398 in sub-F_3:4_ progeny. Progeny testing of homozygous recombinants localized *qNN1* to approximately 18-kb interval between Chr.16G_31103873 and Chr.16G_31122406, which contains 2 putative genes, *Glyma*.*16G150700* and *Glyma*.*16G150800*. Primer sequences of the markers for mapping are listed in S2 Table.

### DNA extraction and PCR assays

Genomic DNA was extracted from soybean leaves by a modified CTAB method [34]. PCR was performed in 25 μL volumes containing 50 ng genomic DNA, 0.5 μL of specific primers (S2 Table), 2.5 μL Ex Tag HS buffer, 2 μL dNTP, 0.125 μL Ex Taq HS DNA polymerase, and distilled, deionized water up to 25 μL. The procedure for PCR amplification were as follows: denaturation at 94°C for 2 min and then 32 cycles of denaturation at 94°C for 30 s, annealing at 50°C to 57°C for 30 s, extension at 72°C for 30 s, and a final extension at 72°C for 10 min. PCR products were digested using corresponding restriction endonucleases in a final volume of 20 μL according to kit protocols. Then, digested products were visualized after electrophoresis on a 3% polyacrylamide gel.

### RNA extraction and qRT-PCR analysis

Total RNA was extracted from various tissues of different plant tissue as described in the text using TRIzol reagent and was reverse transcribed using the M-MLV Reverse Transcriptase kit based on protocols from the supplier. qRT-PCR was performed in 10 μL volumes containing 1 μL of 1:50 diluted cDNA, 0.3 μL of specific primers, 3.4 μL of distilled, deionized water, and 5 μL of Trans Start Top Green qPCR SuperMix using a LightCycler96 PCR system. The elongation factor *EF-1α* gene from soybean (*TefS1*; accession no. X56856) or from tobacco (*NtEF1a*; accession no. AF120093) [35] was used as a reference gene to evaluate relative transcript abundance. Relative transcript abundance was calculated as the ratio of the expression value of the target gene to that of *TefS1* or *NtEF1a* using the 2^-△△CT^ (threshold cycle) method. Primers used for detecting target genes are listed in S3 Table.

### Vector construction and plant transformation

For promoter activity assays, the 2-kb upstream sequence of *GmNN1/FT2a* was amplified from ND and NS genotypes using the primers *proGmNN1/FT2a*-F1/*proGmNN1/FT2a*-R1. The PCR products were cloned into *EcoR*I and *BamH*I sites of the pTF102 vector to create *proGmNN1/FT2a*::*GUS* constructs suing the ClonExpress II One Step Cloning Kit.

For CRISPR/Cas9 gene editing, 2 single-guide RNAs were designed using Primer Design software. Two respective U6 promoters were used for the guide RNA oligonucleotide pair. U6 promoters driving single-guide RNA cassettes were cloned into the pGEL201 vector.

To construct the *GmNN1/FT2a* overexpression or GmNN1/FT2a-GFP plasmid, the ORF of *GmNN1/FT2a* was amplified by PCR using the primers *GmNN1/FT2a*-OE-F/*GmNN1/FT2a*-OE-R and *GmNN1/FT2a*-GFP-F/*GmNN1/FT2a*-GFP-R. Amplified fragments were cloned into *Nco*I and *BamH*I sites of the pFGC5941-p35S vector and *Asc*I sites of the pFGC5941-p35S-GFP vector. All primers are listed in S4 Table.

All constructs for soybean transformation were introduced into *Agrobacterium tumefaciens* strain EHA105 and then transformed into Ws82. In tobacco leaves, recombinant plasmids were introduced into the *A*. *tumefaciens* strain GV3101 and then transiently transferred into tobacco leaves by infiltration. After 2 d, transgenic leaves were harvested for GUS staining and activity analysis.

### GUS staining and fluorometric GUS activity assay

For GUS staining, tobacco leaves were incubated in GUS staining solution containing 50 mm inorganic phosphate-buffered saline (Na_2_HPO_4_-NaH_2_PO_4_ buffer (pH 7.2)), 0.1% (v/v) Triton X-100, 2 mm K_3_Fe(CN)_6_, 2 mm K_4_[Fe(CN)_6_]·3H_2_O, 10 mm EDTA-2Na, and 2 mm 5-bromo-4-chloro-3-indolyl-β-D-GlcA at 37°C overnight. After staining, samples were incubated in 70% (v/v) and then in 100% ethanol until the chlorophyll was completely removed.

For fluorometric GUS assay, protein quantities in transgenic tobacco leaves were first determined based on published methods using bovine serum albumin as a standard [36]. Then, GUS enzyme activity was measured by the fluorescence of 4-methylumbelliferone produced by GUS cleavage of 4-methylumbelliferyl-β-D-glucuronide according to published procedures as described previously [37,38]. GUS activity was calculated as nanomoles of methylumbelliferone per minute per milligram of protein.

### Immunoprecipitation (IP) and immunoblotting

Proteins were extracted in IP buffer (50 mM Tris (pH 8.0), 150 mM NaCl, 1 mM EDTA (pH 8.5), 1% Triton X-100, 1 mM phenylmethylsulfonyl fluoride (PMSF)) containing the complete protease inhibitor mixture. Extracted GmNN1/FT2a-GFP proteins were captured on anti-GFP magarose beads for 1 h at 4°C, washed 3 times with wash buffer (50 mM Tris (pH 8.0), 150 mM NaCl, 1 mM EDTA (pH 8.5), 0.1% Triton X-100), and transferred into a new tube and incubated at 95°C for 10 min before elution with SDS sample buffer (0.5 M Tris-HCl (pH 6.8), 10% SDS, 20% glycerol, 2% β-mercaptoethanol, 1% (w/v) bromophenol blue).

For the detection of GmNN1/FT2a-GFP proteins, eluted samples were separated by SDS-PAGE in an 8% agarose gel and then transferred to polyvinylidene fluoride (PVDF) membranes. Membranes were blocked in 5% skim milk for 1 h at room temperature prior to incubating with the primary anti-GFP antibodies overnight at 4°C. Horseradish peroxidase (HRP)-conjugated secondary antibodies were then added at 1:5,000 dilutions for 45 min.

### Protein–protein interaction assays

For the yeast 2-hybrid assay, the GmNN1/FT2a ORF was amplified using the primers GmNN1/FT2a-F1/GmNN1/FT2a-R1 and subcloned into the prey plasmid pGADT7. The ORF of GmNFYA-C was amplified using the primers GmNFYA-C-F1/GmNFYA-C-R1 and subcloned into the bait plasmid pGBKT7. Yeast 2-hybrid assays were performed using the *Saccharomyces cerevisiae* strain AH109. Interaction was determined by co-transforming the prey and bait vectors into yeast and growing the yeast colonies on SD-His-Trp-Leu in 30 mM 3AT plates. Yeast transformation and growth were performed as described in the Yeast Protocols Handbook.

For semi-in vivo pull-down assays, the ORF of *GmNFYA-C* was amplified with the primers MYC-GmNFYA-C-F1/MYC-GmNFYA-C-R1 and then inserted into the pENTR 1A Entry vector. Entry clones were subcloned into Gateway compatible pEarleyGate203 (35S-MYC-Gene) vectors using the LR reaction. Both the MYC-tagged GmNFYA-C (MYC-NFYA-C) and GmNN1/FT2a-GFP were introduced into *A*. *tumefaciens* strain GV3101 and then transiently co-expressed in tobacco leaves using the same IP method as descried above to enrich the FT2a-GFP protein. Eluted samples were analyzed on immunoblots using anti-GFP (diluted 1:1,000) and anti-MYC antibodies (diluted 1:5,000).

For in vivo bimolecular fluorescence complementation (BiFC) assays, the ORFs of *GmNN1/FT2a*, *GmNYFA-C*, *GmPHR9*, and *GmSPX3* were amplified using the primers as listed in S4 Table prior to separately inserting them into the vectors pCAMBIA1300-nYFP and pCAMBIA1300-cYFP. pCAMBIA1300-nYFP is the vector for N-terminal fusion to yellow fluorescent protein (nYFP), and pCAMBIA1300-cYFP is the vector for C-terminal fusion to YFP (cYFP). Among them, GmPHR9 was reported to be interacted with GmSPX3 in yeast assay [39], and here, leaf epidermal cells of *N*. *benthamiana* were co-transformed with GmPHR9-cYFP and GmNN1/FT2a-nYFP or GmNFYA-C-cYFP and GmSPX3-nYFP was used as control according to previous description [40]. All of the different plasmid combinations were introduced into *A*. *tumefaciens* strain GV3101 and then transiently transferred into tobacco leaves through infiltration. After 2 d, transgenic leaves were examined and imaged using a confocal laser scanning microscope.

### Chromatin immunoprecipitation (Ch-IP)-qPCR assay

For ChIP-qPCR analysis, seeds of the stable transgenic plant harboring GmNN1/FT2a-GFP were surface sterilized in 3% H_2_O_2_ for 1 min, rinsed with distilled water, and germinated in sand for 5 d. Uniform seedlings with unfolded cotyledons were infected on the hypocotyl with *A*. *rhizogenes* strain K599 carrying the pGEL201 vector to generate *nfya-c* mutant hairy roots. When the emerged hairy roots were approximately 10-cm long, the main roots were removed and the hairy roots were collected for DNA extraction and sequencing. Selected transgenic soybean composite plants were inoculated with rhizobial strain BXYD3 and then grown in sand irrigated with low-N nutrient solution for 5 d. Four biological replicates of samples were collected, and each replicate was collected from 3 independent hairy roots. A 2-g aliquot of tissue was then fixed by formaldehyde cross-lining, and the ChIP-qPCR assay procedures were performed as described previously [41]. Target chromatin was immunoprecipitated using anti-GFP magnetic beads. Enrichment of DNA fragments was determined by qRT-PCR analysis. The fold-enrichment was calculated by normalizing target fragment against internal reference gene *GmELF1B* (Glyma.02G276600). The relevant primer sequences are given in S5 Table.

### Measurement of N concentration

For N determination, shoots were dried at 105°C for 30 min, followed by heating at 65°C until completely dry. Samples were ground into powder and digested by H_2_SO_4_ [42]. N concentrations were measured by the sodium hypochlorite salicylic acid colorimetric method using a continuous flow analyzer running samples at a wavelength of 660 nm and a temperature of 40°C. Colorimetric signals were recorded using FlowAccess as recommended by manufacturer.

### Data analysis

For phenotypic evaluation, at least 4 individual plants were analyzed and 2 independent experiments were performed. For expression analyses using qRT-PCR, at least 3 individual plants were pooled per tissue sample, and at least 3 qRT-PCR reactions (technical replicates) for each sample were performed. The exact number of replicates (*n*) is stated in the figure legends. Mean values for each measured parameter were compared using 2-sided Student *t* tests or Duncan’s multiple range comparison tests when appropriate.

## Supporting information

S1 Fig Distribution of variation between the ND and NS genotypes across soybean chromosomes. 1–4 represent highly heterozygous sites. Data underlying the graphs in the figure can be found in S2 Data. ND, N deficient; NS, N sufficient.
(TIF)

S2 Fig *GmNN1/FT2a* expression analysis. **(A)** GUS staining of tobacco leaves harboring different *GmNN1/FT2a* promoters constructed from ND and NS plant materials. **(B)** Relative expression of the *GUS* gene (*n* = 7). **(C)** Quantitative GUS activity analysis of the transgenic tobacco leaves by fluorimetric assay (*n* = 7). **(D)** Relative expression of *GmNN1/FT2a* in NILs (*n* = 3). Three independent lines of each NIL were generated, and 3 biological replicates for each line were harvested for qRT-PCR analysis. All data are given as mean ± SD. Asterisks denote significance of differences (threshold *P* = 0.05) according to Student *t* tests; **P < 0.001; ***P < 0.001. Data underlying the graphs in the figure can be found in S2 Data. ND, N deficient; NIL, near-isogenic line; NS, N sufficient; qRT-PCR, quantitative reverse transcription PCR.
(TIF)

S3 Fig Genotype of the *Gmnn1/ft2a* mutants edited by the CRISPR/Cas9 system. **(A)** The target site of CRISPR/Cas9 editing in the first exon led to a 1-bp insertion when compared with the WT plant sequence. **(B)** The target site of CRISPR/Cas9 editing in the first exon led to 1-bp deletion compared with the WT sequence. WT, wild type.
(TIF)

S4 Fig Phenotype and molecular identification of *GmNN1/FT2a* in stable transgenic soybean plants. **(A)** Flowering phenotype as affected by overexpression or knockout of *GmNN1/FT2a*. **(B)** Relative expression of *GmNN1/FT2a* in leaves (*n* = 4). **(C)** Flowering time in various plants (*n* = 8). All data are given as mean ± SD. Different letters denote significant differences (*P* < 0.05) according to Duncan’s multiple range comparison tests. Data underlying the graphs in the figure can be found in S2 Data.
(TIF)

S5 Fig Comparison of nodulation traits between ND and NS soybean materials. **(A)** Phenotype of nodules. Scale bars, 2 cm. **(B)** Nodule number per plant. **(C)** Nodule dry weight. Soybean plants inoculated with rhizobia were grown in hydroponics for 21 d prior to harvesting nodules for trait analysis. All data are given as mean from 2 independent experiments ± SD (*n* = 15). Asterisks denote significance of differences (threshold *P* = 0.05) according to Student *t* tests; ***P < 0.001. Data underlying the graphs in the figure can be found in S2 Data. ND, N deficient; NS, N sufficient.
(TIF)

S6 Fig Expression of *GmNN1/FT2a* in various tissues of soybean plants. Ws82 was inoculated with rhizobial BXYD3 and transplanted into low-N nutrient solution for 14 d. Then, roots, nodules, stems, YL, and OL were separately harvested for qRT-PCR analysis. Data underlying the graphs in the figure can be found in S2 Data. N, nitrogen; OL, old leaf; qRT-PCR, quantitative reverse transcription PCR; YL, young leaf.
(TIF)

S7 Fig Plant dry weight and N content assays for grafts between *Gmnn1*/*ft2a* mutant and Ws82 at 21 dai. **(A)** Plant dry weight (*n* = 10). **(B)** N content (*n* = 7). *Gmnn1*/*ft2a* mutant: stable transgenic soybean with knockout of *GmNN1/FT2a*; Ws82: WT control. Data are given as mean ± SD. Different letters denote significant differences (*P* < 0.05) according to Duncan’s multiple range comparison tests. Data underlying the graphs in the figure can be found in S2 Data. dai, day after inoculation; N, nitrogen; WT, wild type.
(TIF)

S8 Fig GmNN1/FT2a interacted with GmNYFA-C in the BiFC assay. Leaf epidermal cells of *N*. *benthamiana* were co-transformed with GmPHR9-cYFP and GmNN1/FT2a-nYFP or GmNFYA-C-cYFP and GmSPX3-nYFP was used as control according to previous description [40]. BF, bright field; Merge, overlay of the YFP and bright field images.
(TIF)

S9 Fig Genotype of the *nfya-c* mutant edited by the CRISPR/Cas9 system from soybean hairy roots. The target site of CRISPR/Cas9 editing in the first exon led to a 2-bp deletion when compared with the WT plant sequence. WT, wild type.
(TIF)

S10 Fig Nodule dry weight on grafts. Nodules were harvested at 21 dai. *Gmnn1/ft2a* mutant: stable transgenic soybean with knockout of *GmNN1/FT2a*; *nfya-c*: stable transgenic soybean with knockout of *GmNFYA-C*; Ws82: WT control. Data are given as mean ± SD (*n* = 6–8). Different letters denote significant differences (*P* < 0.05) according to Duncan’s multiple range comparison tests. Data underlying the graphs in the figure can be found in S2 Data. dai, day after inoculation; WT, wild type.
(TIF)

S1 Table Variation between the ND and NS across soybean chromsomes. 0|0 and 1|1 from both ND and NS represent reference and mutant single-nucleotide polymorphisms, respectively. ND, N deficient; NS, N sufficient.
(XLS)

S2 Table Gene specific primers used for *GmNN1/FT2a* fine mapping. (XLSX)

S3 Table Gene-specific primers used for qRT-PCR analysis. (XLSX)

S4 Table Gene-specific primers used for vector constructs. (XLSX)

S5 Table Primer sequences used for ChIP-qPCR assay. (XLSX)

S1 Raw Images Raw images of the western blot shown in Figs 4H, 4J and 5C. (PDF)

S1 Data Data underlying Figs 1B, 1C, 1E, 1J, 1K, 2B, 2C, 2E, 2F, 2H, 2I, 3B, 3C, 3D, 3E, 3F, 4B, 4D, 4E, 4F, 4G, 5A, 5B, 6E and 6G. (XLSX)

S2 Data Data underlying S1, S2B–S2D, S4B, S4C, S5B, S5C, S6, S7A, S7B and S10 Figs. (XLSX)

## Abbreviations:

AON: autoregulation of nodulation
BiFC: bimolecular fluorescence complementation
dai: day after rhizobium inoculation
GUS staining: β-Glucuronidase staining
HRP: horseradish peroxidase
IP: immunoprecipitation
N: nitrogen
ND: N deficient
NIL: near-isogenic line
NS: N sufficient
PVDF: polyvinylidene fluoride
qRT-PCR: quantitative reverse transcription PCR
QTL: quantitative trait locus
SNF: symbiotic nitrogen fixation
WT: wild type
