# Supplementary figures and images for: Shoot-to-root translocated GmNN1/FT2a triggers nodulation and regulates soybean nitrogen nutrition
Source: PLoS Biol. 2022 Aug 15;20(8):e3001739. doi: 10.1371/journal.pbio.3001739 (PMC9410562; doi:10.1371/journal.pbio.3001739)

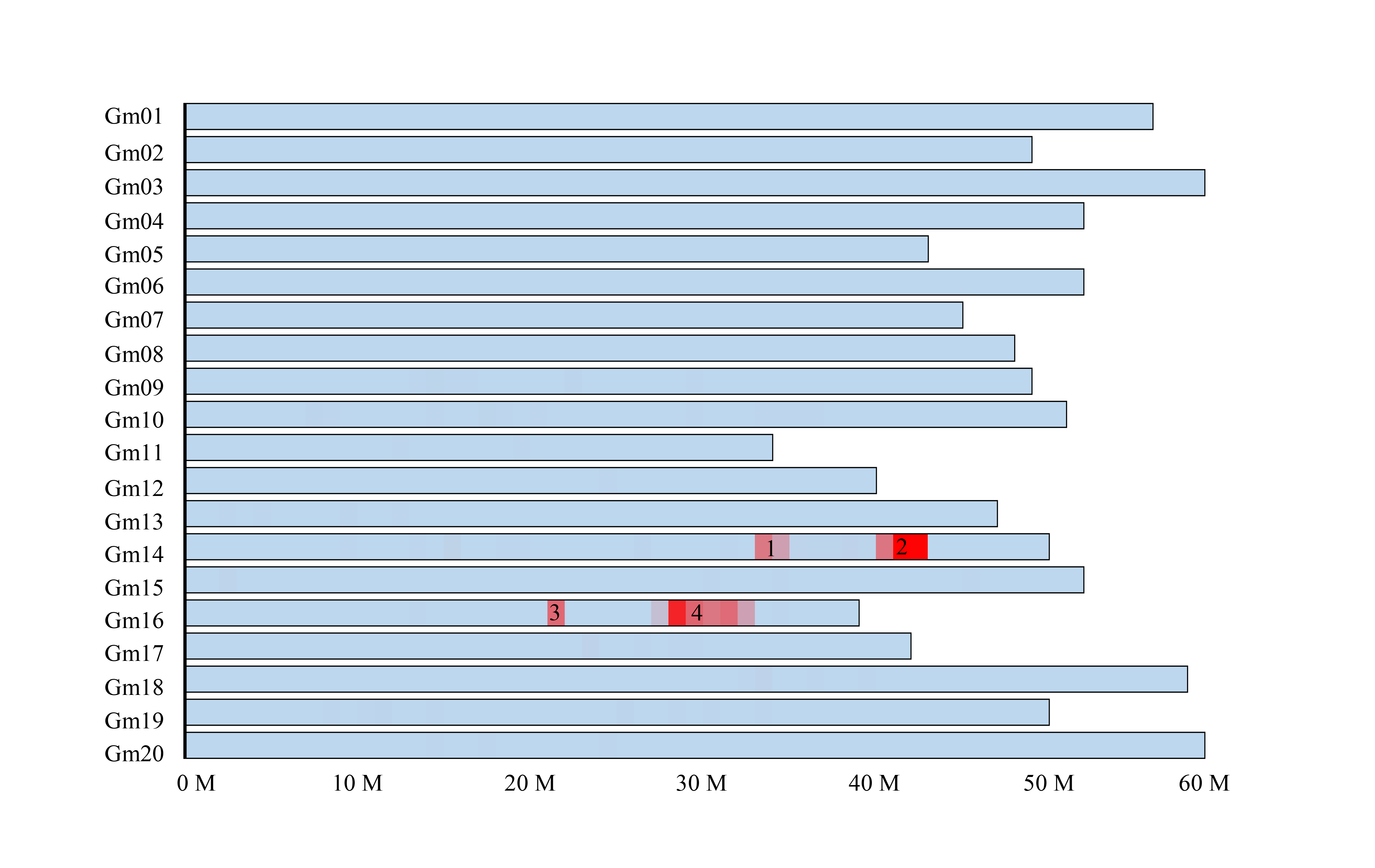

Supplement: S1 Fig — 1–4 represent highly heterozygous sites. Data underlying the graphs in the figure can be found in S2 Data. ND, N deficient; NS, N sufficient. (TIF) [file pbio.3001739.s001.tif]

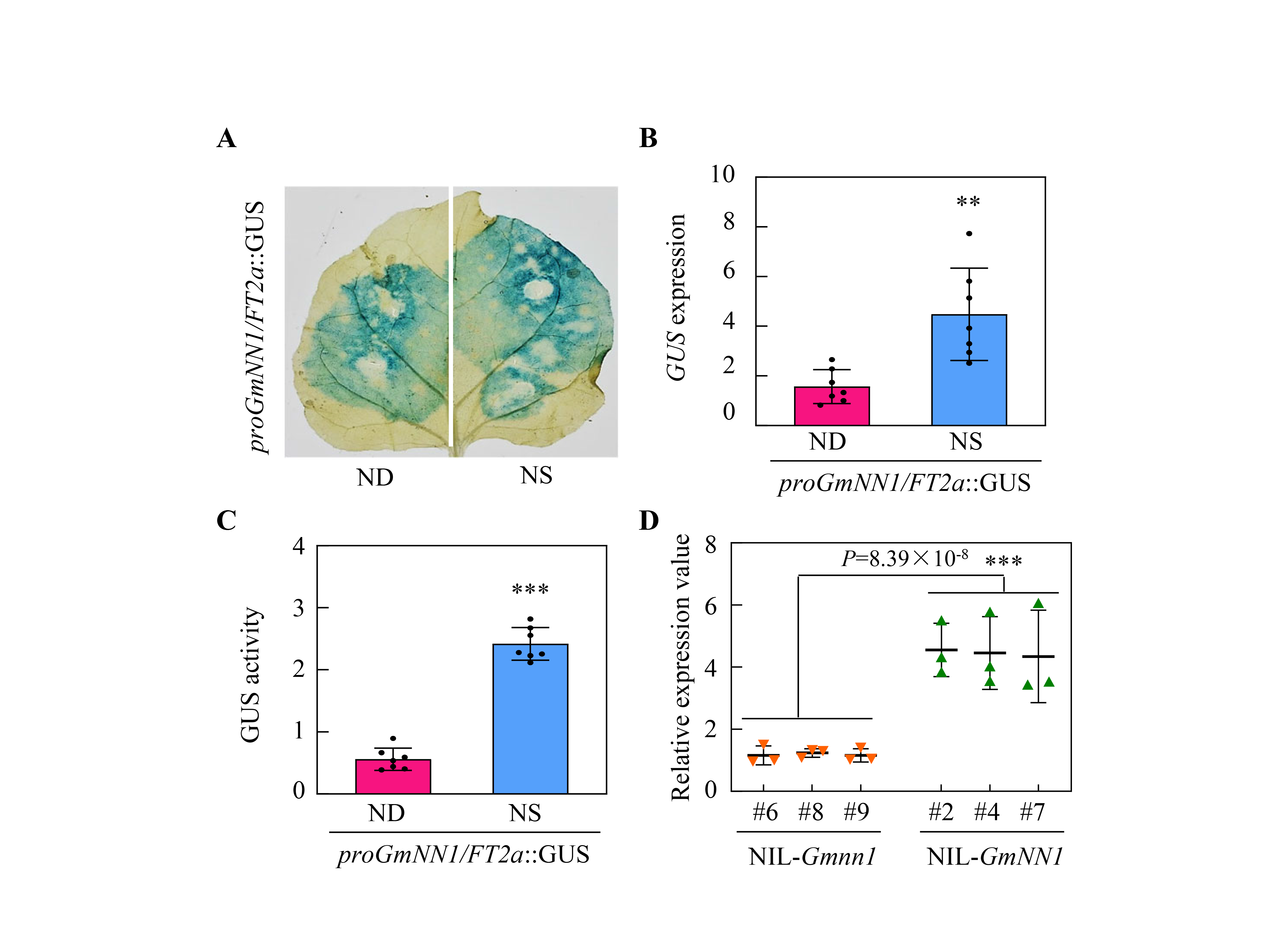

Supplement: S2 Fig — (A) GUS staining of tobacco leaves harboring different GmNN1/FT2a promoters constructed from ND and NS plant materials. (B) Relative expression of the GUS gene (n = 7). (C) Quantitative GUS activity analysis of the transgenic tobacco leaves by fluorimetric assay (n = 7). (D) Relative expression of GmNN1/FT2a in NILs (n = 3). Three independent lines of each NIL were generated, and 3 biological replicates for each line were harvested for qRT-PCR analysis. All data are given as mean ± SD. Asterisks denote significance of differences (threshold P = 0.05) according to Student t tests; **P < 0.001; ***P < 0.001. Data underlying the graphs in the figure can be found in S2 Data. ND, N deficient; NIL, near-isogenic line; NS, N sufficient; qRT-PCR, quantitative reverse transcription PCR. (TIF) [file pbio.3001739.s002.tif]

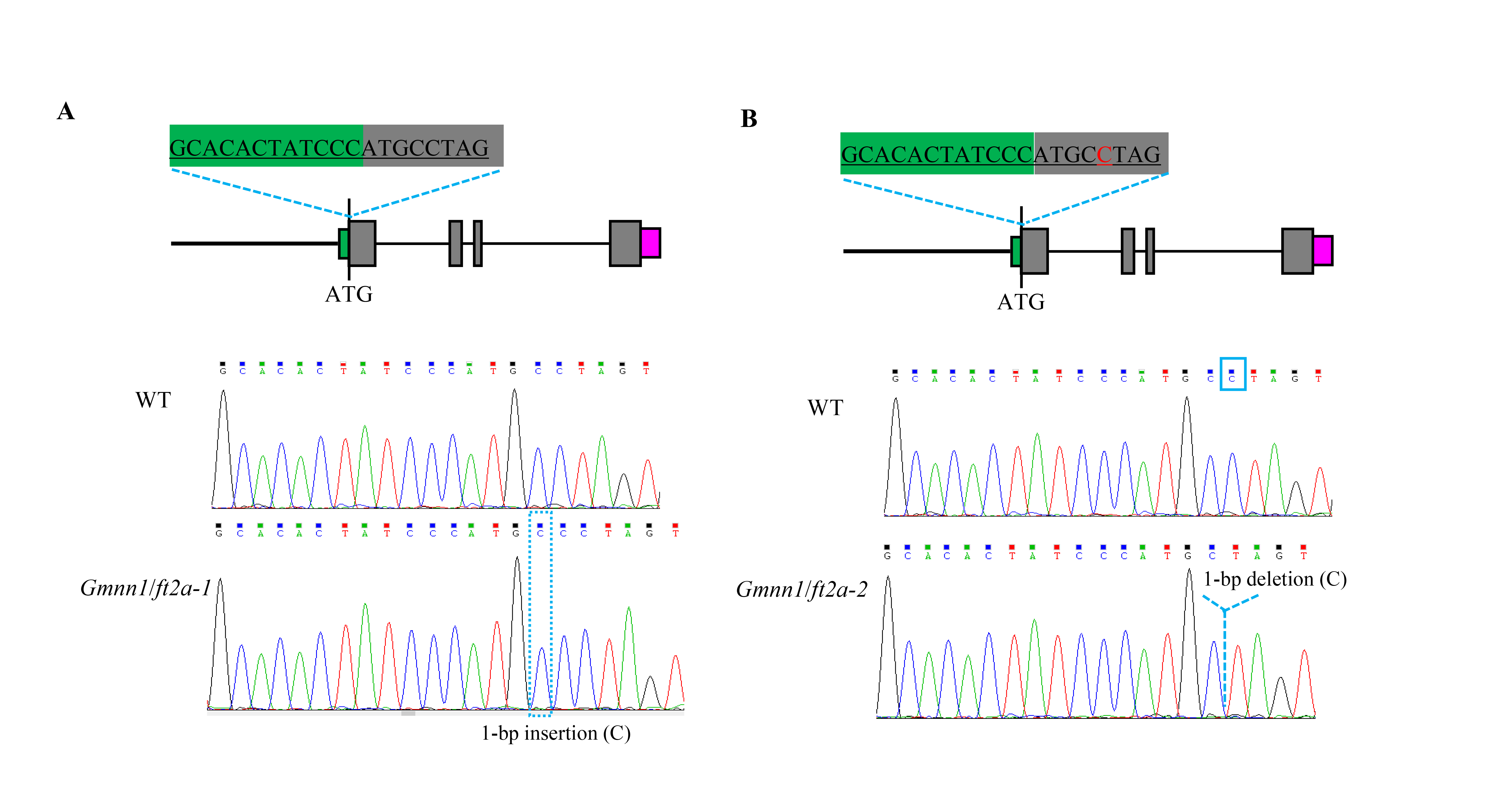

Supplement: S3 Fig — (A) The target site of CRISPR/Cas9 editing in the first exon led to a 1-bp insertion when compared with the WT plant sequence. (B) The target site of CRISPR/Cas9 editing in the first exon led to 1-bp deletion compared with the WT sequence. WT, wild type. (TIF) [file pbio.3001739.s003.tif]

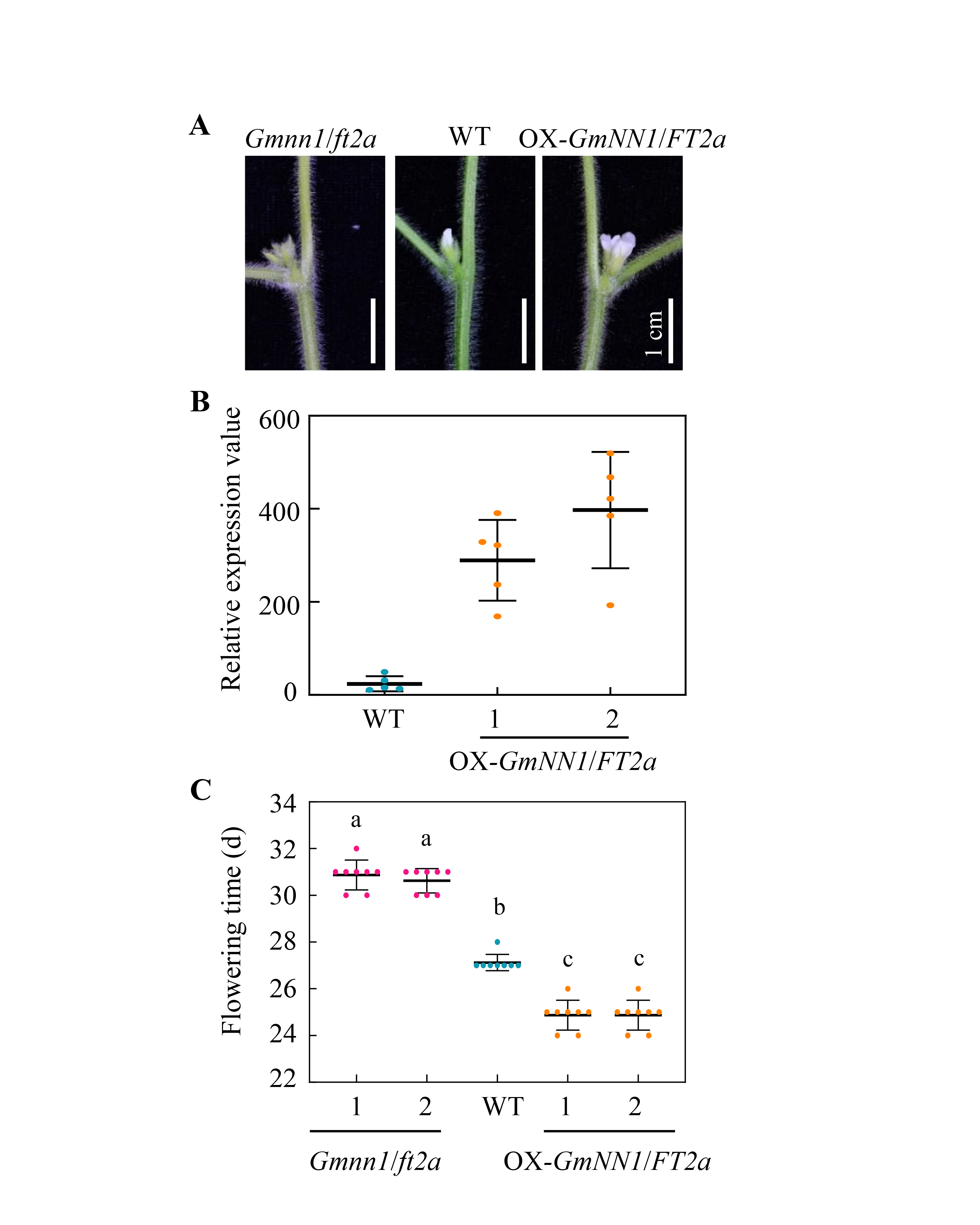

Supplement: S4 Fig — (A) Flowering phenotype as affected by overexpression or knockout of GmNN1/FT2a. (B) Relative expression of GmNN1/FT2a in leaves (n = 4). (C) Flowering time in various plants (n = 8). All data are given as mean ± SD. Different letters denote significant differences (P < 0.05) according to Duncan’s multiple range comparison tests. Data underlying the graphs in the figure can be found in S2 Data. (TIF) [file pbio.3001739.s004.tif]

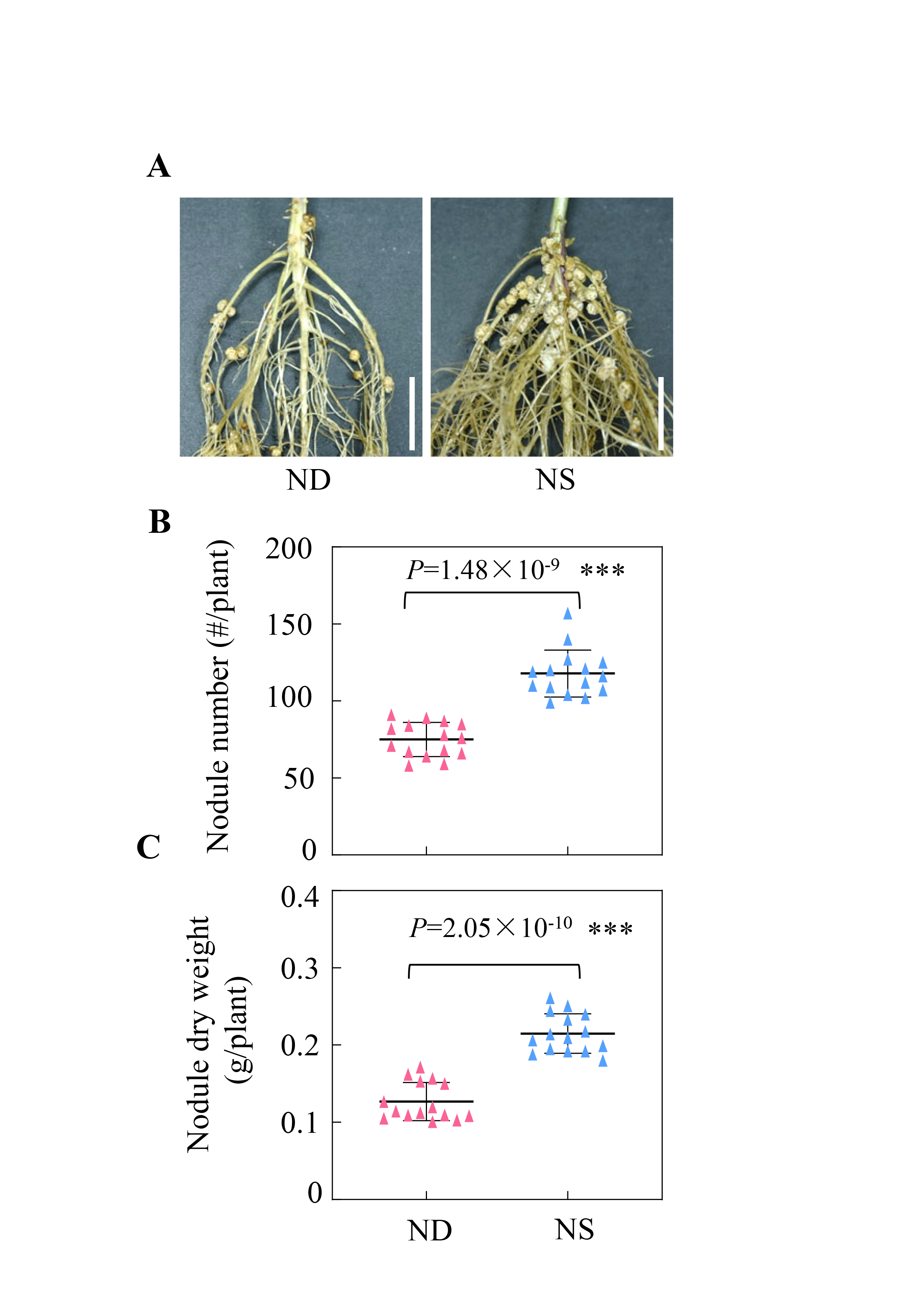

Supplement: S5 Fig — (A) Phenotype of nodules. Scale bars, 2 cm. (B) Nodule number per plant. (C) Nodule dry weight. Soybean plants inoculated with rhizobia were grown in hydroponics for 21 d prior to harvesting nodules for trait analysis. All data are given as mean from 2 independent experiments ± SD (n = 15). Asterisks denote significance of differences (threshold P = 0.05) according to Student t tests; ***P < 0.001. Data underlying the graphs in the figure can be found in S2 Data. ND, N deficient; NS, N sufficient. (TIF) [file pbio.3001739.s005.tif]

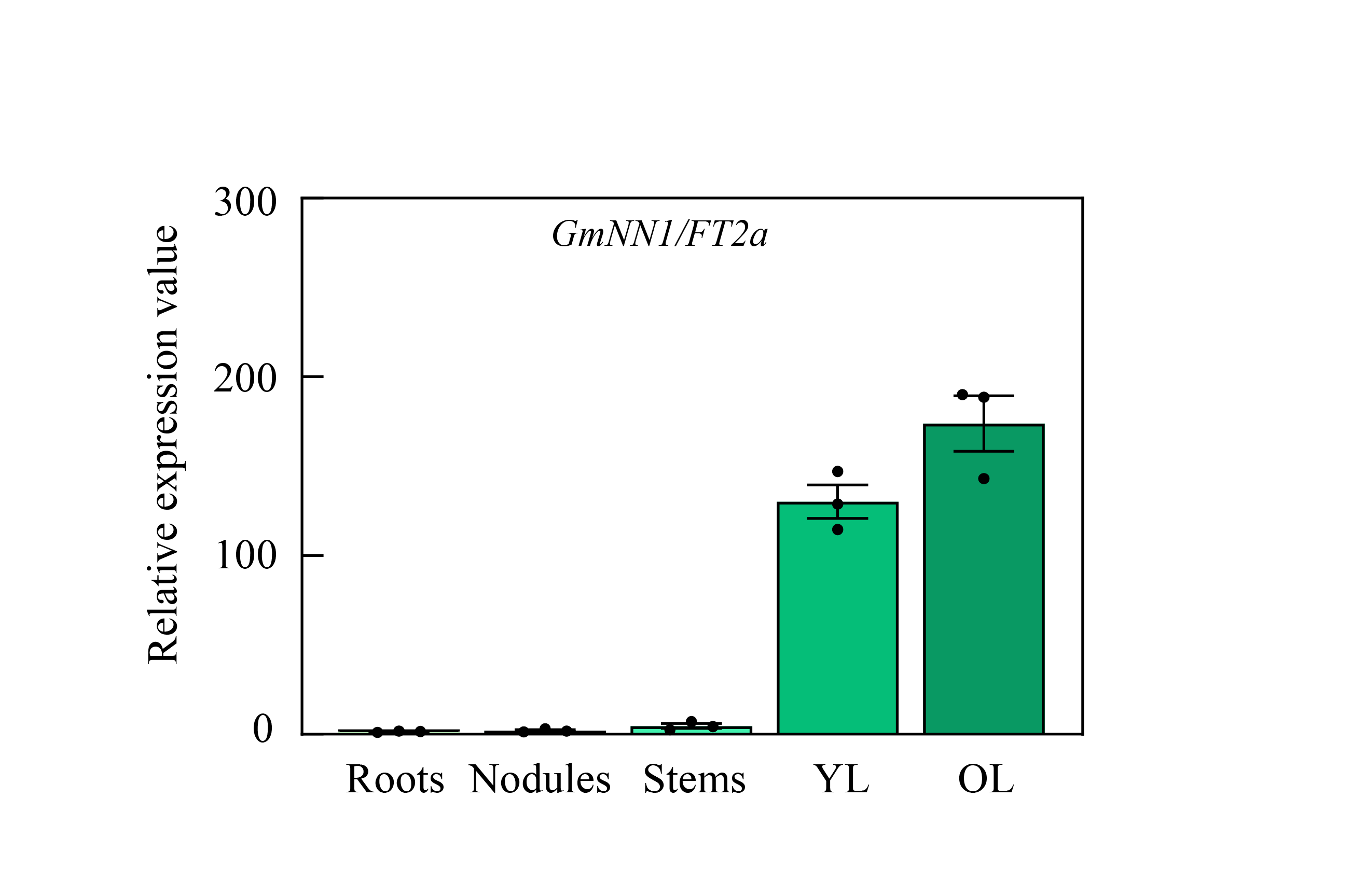

Supplement: S6 Fig — Ws82 was inoculated with rhizobial BXYD3 and transplanted into low-N nutrient solution for 14 d. Then, roots, nodules, stems, YL, and OL were separately harvested for qRT-PCR analysis. Data underlying the graphs in the figure can be found in S2 Data. N, nitrogen; OL, old leaf; qRT-PCR, quantitative reverse transcription PCR; YL, young leaf. (TIF) [file pbio.3001739.s006.tif]

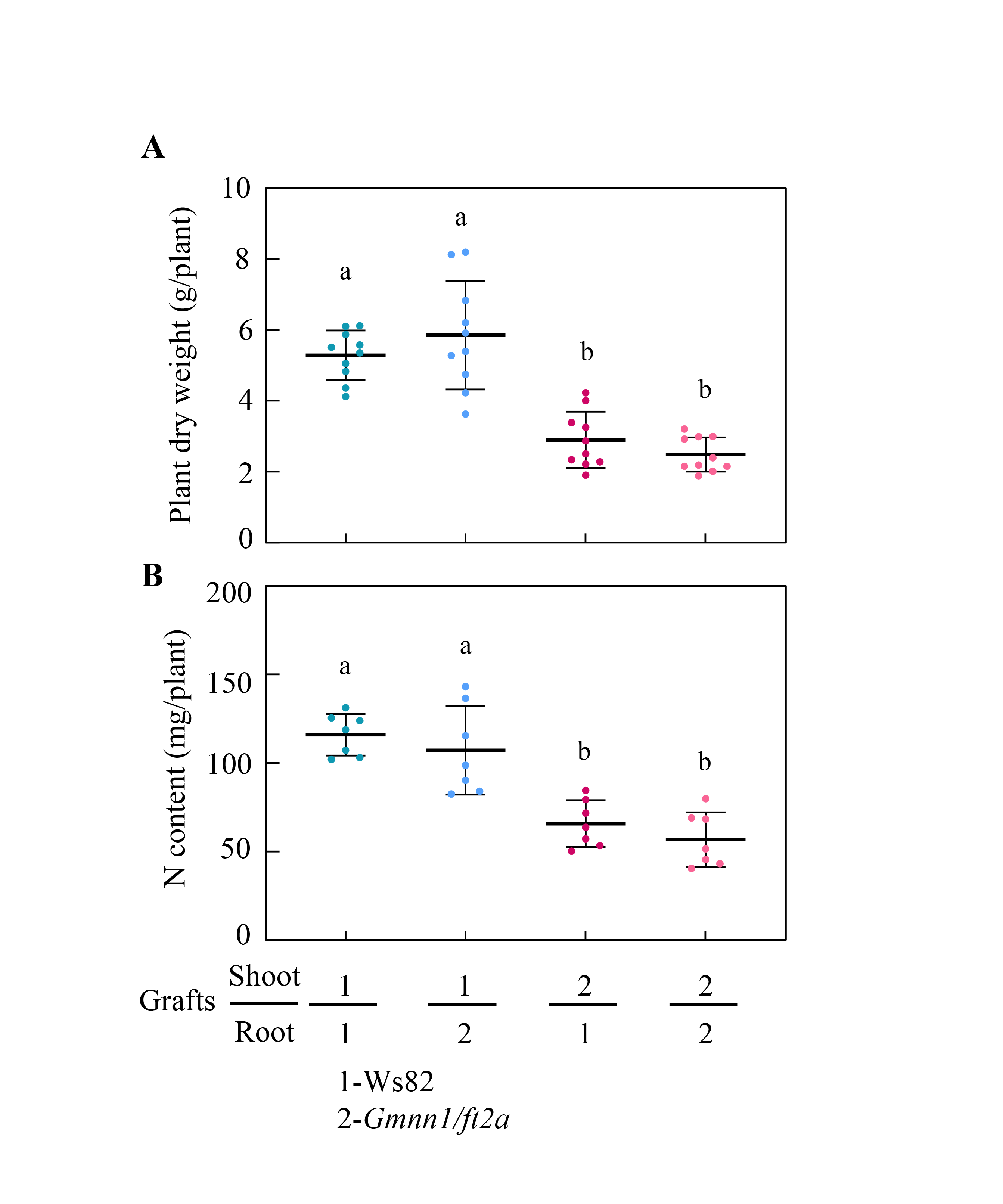

Supplement: S7 Fig — (A) Plant dry weight (n = 10). (B) N content (n = 7). Gmnn1/ft2a mutant: stable transgenic soybean with knockout of GmNN1/FT2a; Ws82: WT control. Data are given as mean ± SD. Different letters denote significant differences (P < 0.05) according to Duncan’s multiple range comparison tests. Data underlying the graphs in the figure can be found in S2 Data. dai, day after inoculation; N, nitrogen; WT, wild type. (TIF) [file pbio.3001739.s007.tif]

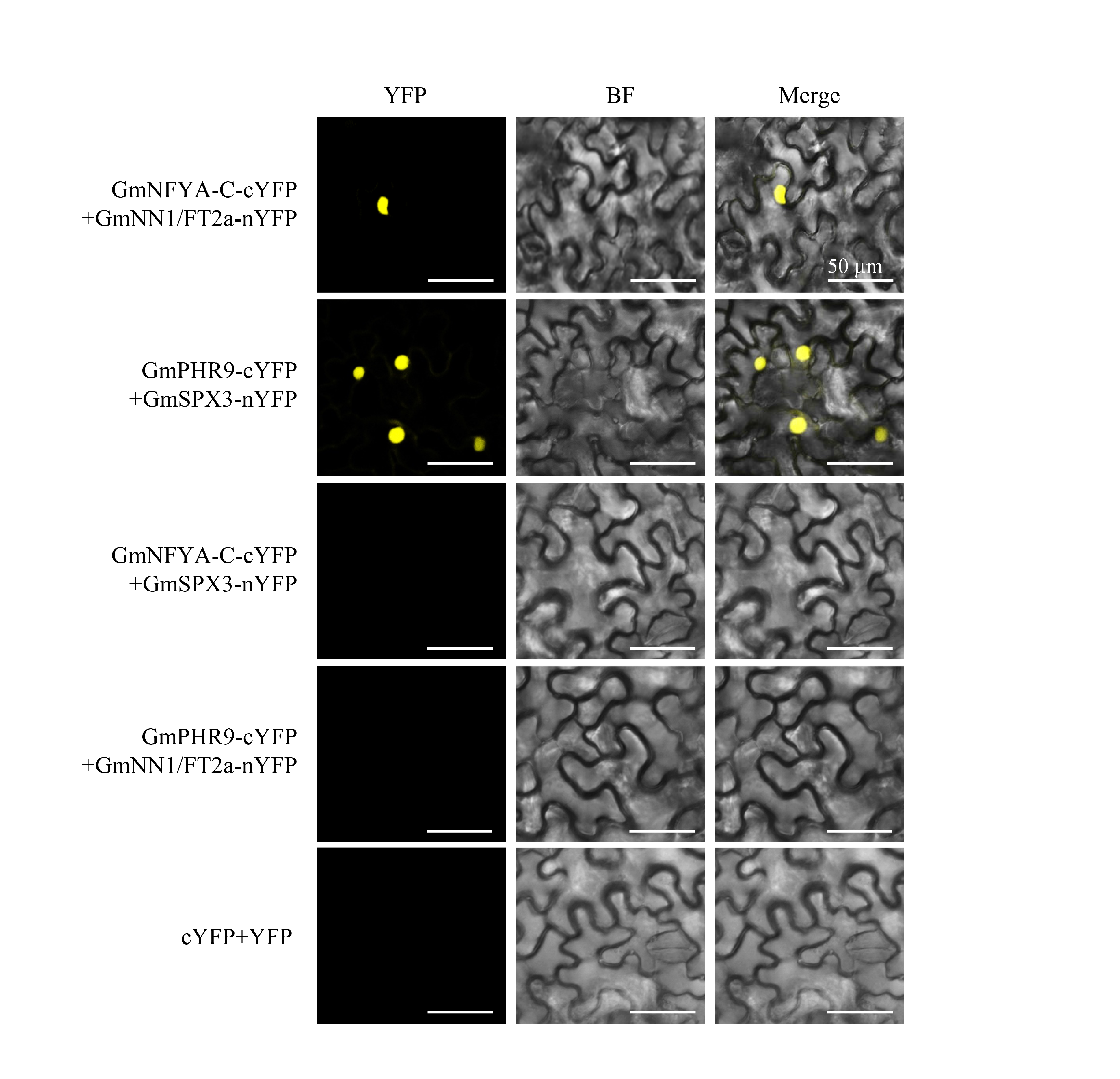

Supplement: S8 Fig — Leaf epidermal cells of N. benthamiana were co-transformed with GmPHR9-cYFP and GmNN1/FT2a-nYFP or GmNFYA-C-cYFP and GmSPX3-nYFP was used as control according to previous description [40]. BF, bright field; Merge, overlay of the YFP and bright field images. (TIF) [file pbio.3001739.s008.tif]

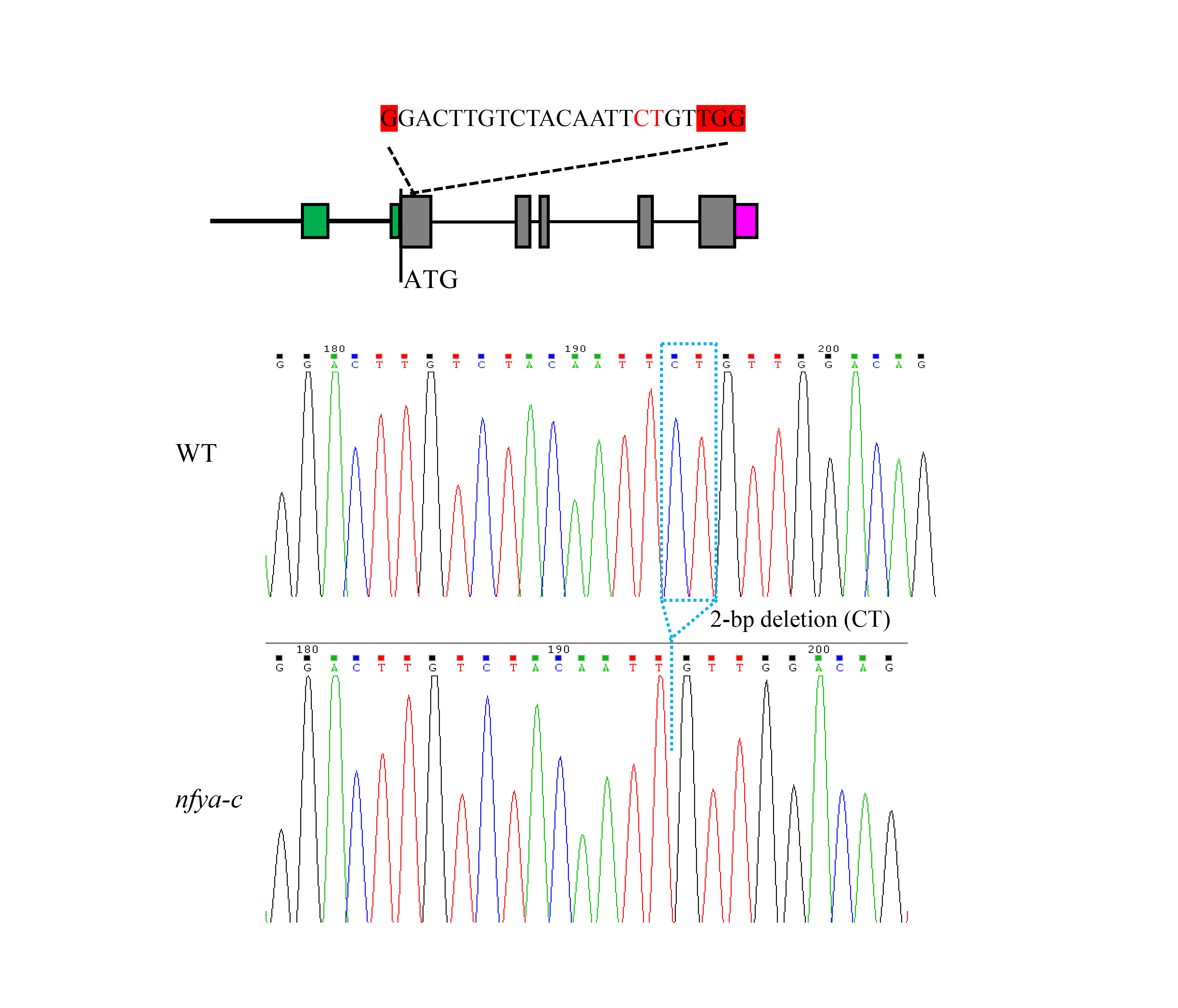

Supplement: S9 Fig — The target site of CRISPR/Cas9 editing in the first exon led to a 2-bp deletion when compared with the WT plant sequence. WT, wild type. (TIF) [file pbio.3001739.s009.tif]

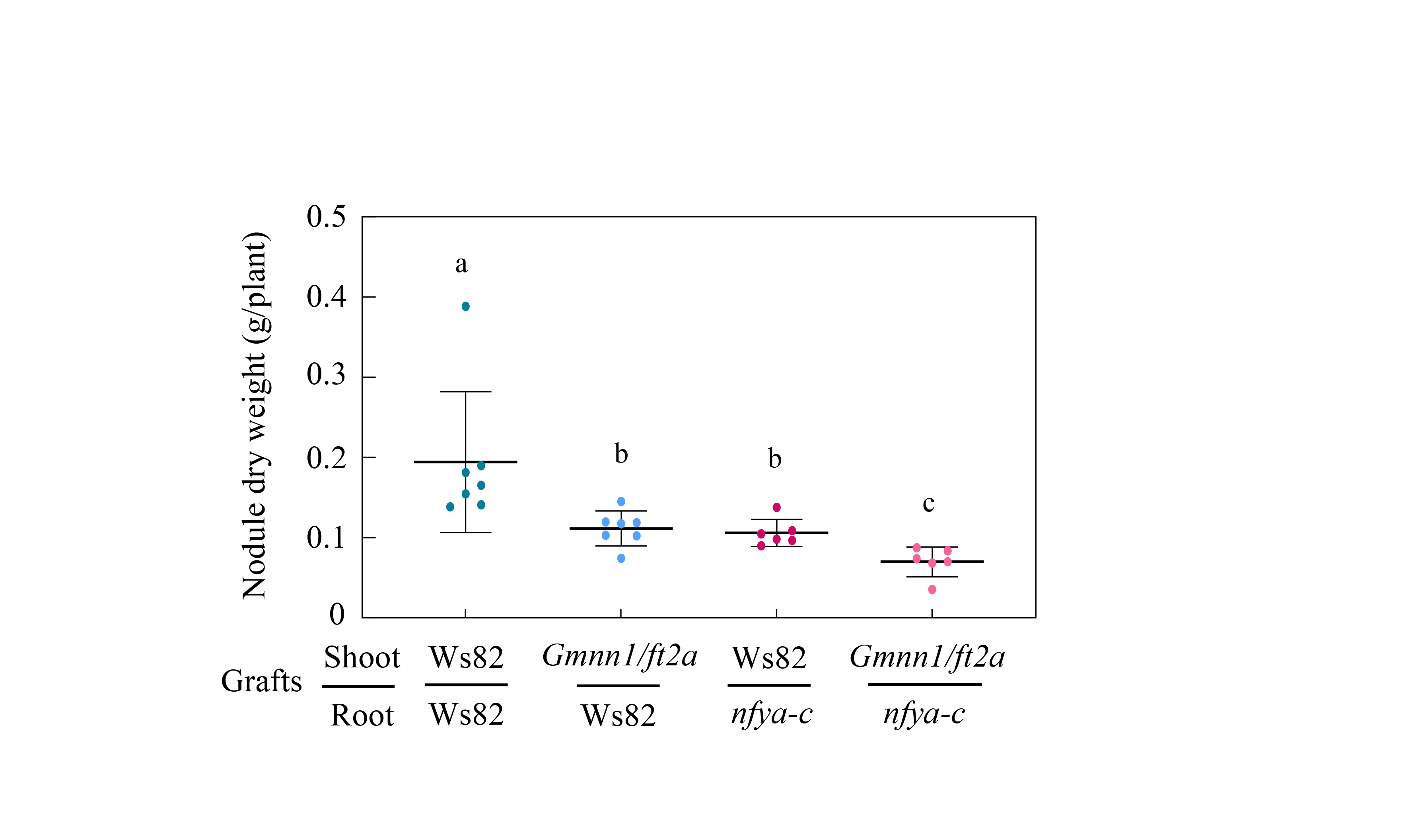

Supplement: S10 Fig — Nodules were harvested at 21 dai. Gmnn1/ft2a mutant: stable transgenic soybean with knockout of GmNN1/FT2a; nfya-c: stable transgenic soybean with knockout of GmNFYA-C; Ws82: WT control. Data are given as mean ± SD (n = 6–8). Different letters denote significant differences (P < 0.05) according to Duncan’s multiple range comparison tests. Data underlying the graphs in the figure can be found in S2 Data. dai, day after inoculation; WT, wild type. (TIF) [file pbio.3001739.s010.tif]
