# Supplementary material for: Shoot-to-root translocated GmNN1/FT2a triggers nodulation and regulates soybean nitrogen nutrition
Source: PLoS Biol. 2022 Aug 15;20(8):e3001739. doi: 10.1371/journal.pbio.3001739 (PMC9410562; doi:10.1371/journal.pbio.3001739)
Supplement: S1 Raw Images — (PDF) [file pbio.3001739.s016.pdf]

Fig. 4H

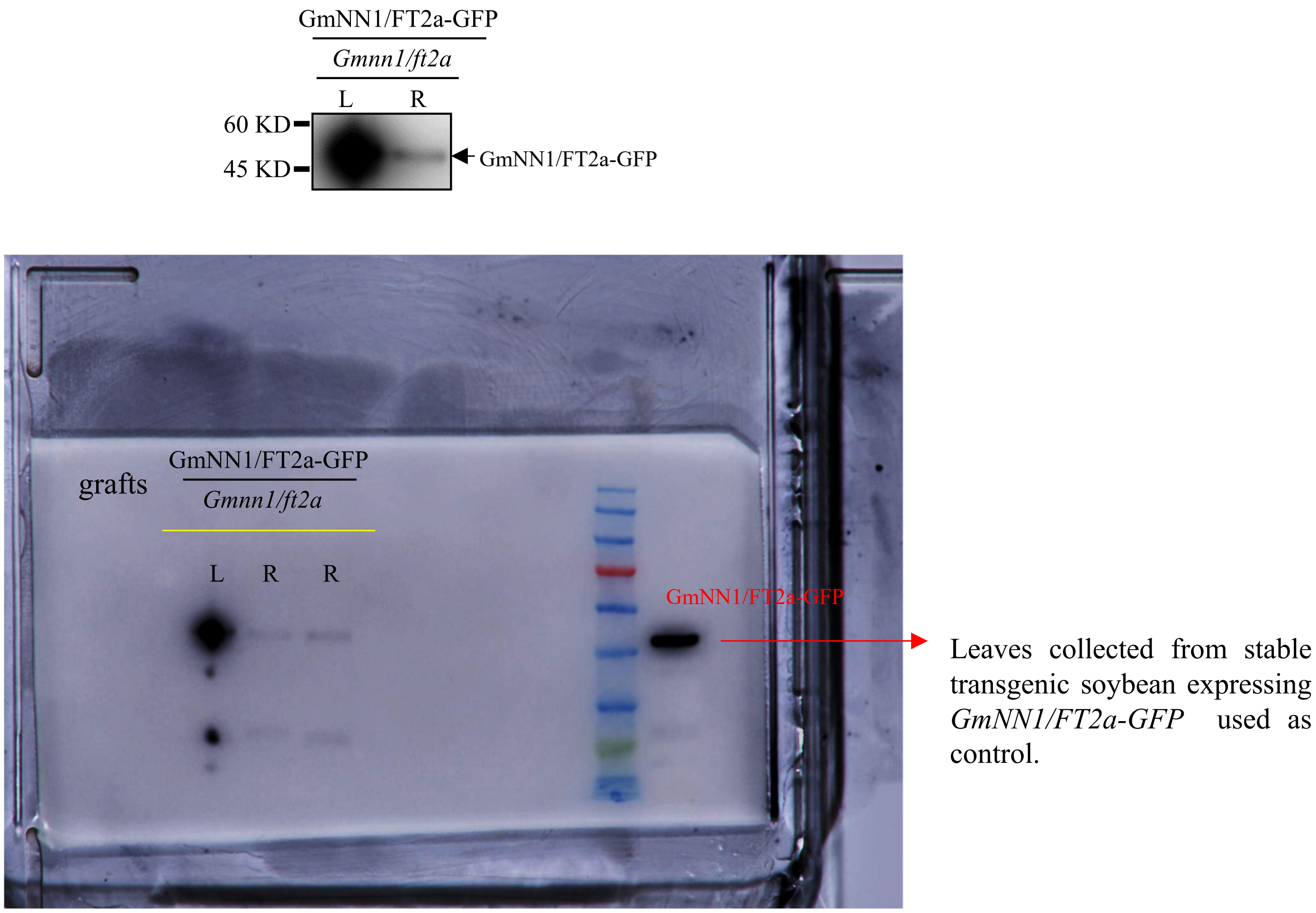

Immunological detection of GmNN1/FT2a-GFP in leaves (L) and roots (R) of grafts at 5 dai. Scions and rootstocks were stable transgenic soybean plants harboring GmNN1/FT2a-GFP and a knock out of *GmNN1/FT2a* (*Gmnn1/ft2a*), respectively.

Fig. 4J

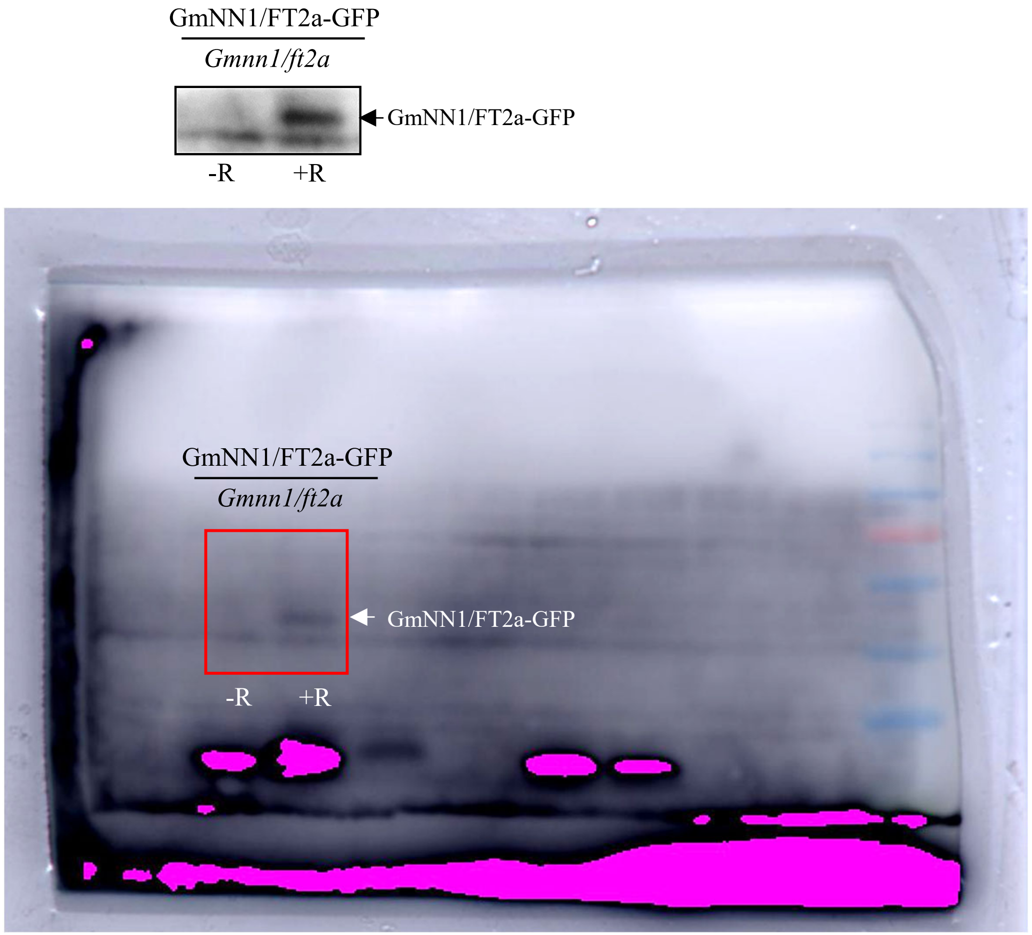

Immunological detection of GmNN1/FT2a-GFP from split roots five days after inoculation with (+R) or without (-R) rhizobia.

Fig. 5C

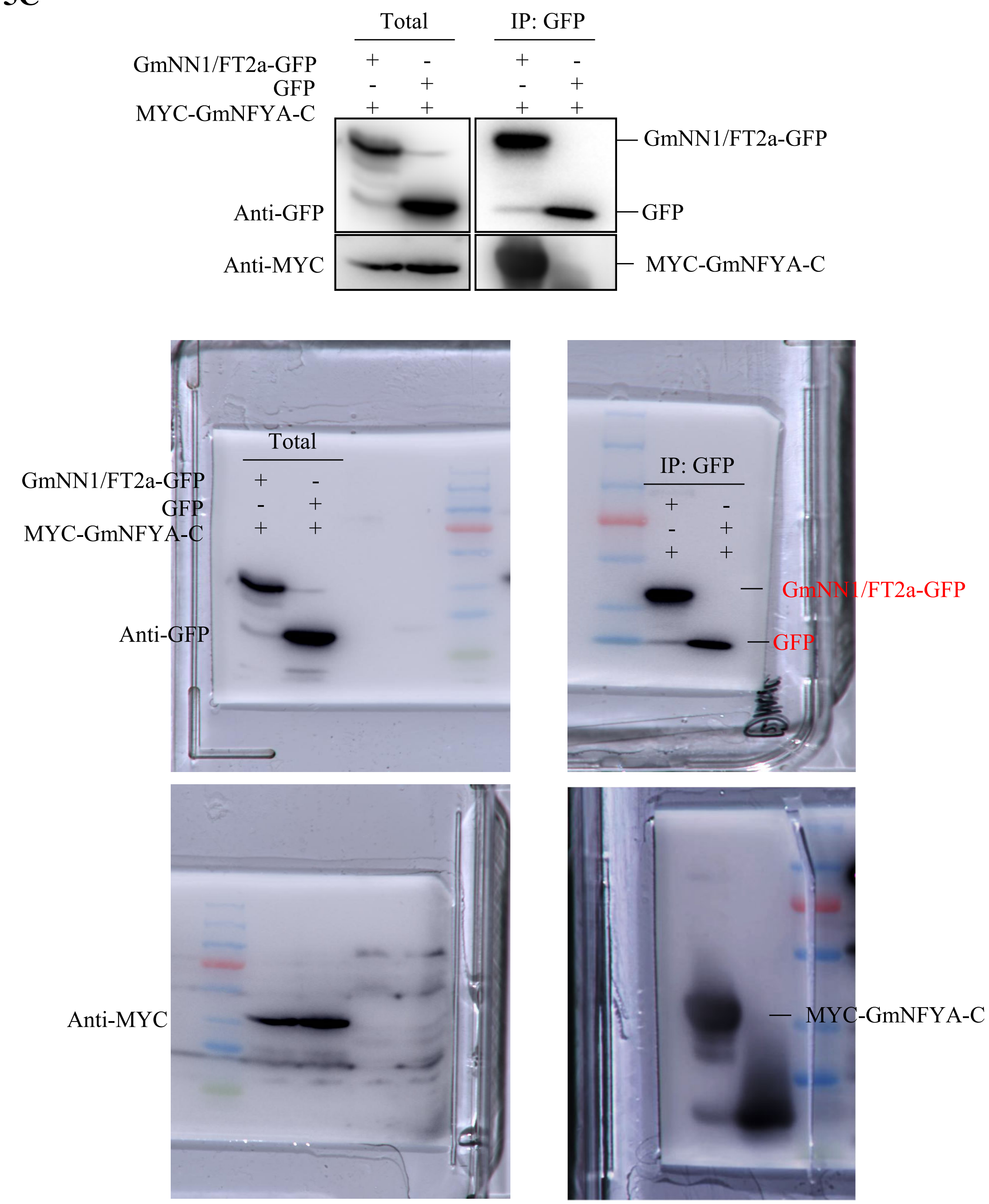

GmNN1/FT2a interacting with GmNFYA-C in *vitro* pull-down assays.
